# MoR–Swin: Efficient Vision Transformer Using Mixture of Recursions

**DOI:** 10.3390/jimaging12080377

**Published:** 2026-08-12

**Authors:** Yongbao Ai, Tianxiang Gao, Zhipeng Lin, Longqi Yang, Qingyu Chang

**Affiliations:** Academy of Military Science, Institute of Military Intelligence, Beijing 100091, China; gaotxnudta@163.com (T.G.); linzhipeng13@nudt.edu.cn (Z.L.); seriousthing@yeah.net (L.Y.); changqingyu@mail.ecust.edu.cn (Q.C.)

**Keywords:** Swin Transformer, mixture of recursions, adaptive computation, parameter efficiency

## Abstract

Vision Transformers, especially Swin Transformer, have become default backbones for various vision tasks but suffer from high memory consumption and training costs. This letter proposes MoR–Swin, a novel architecture that integrates Mixture of Recursions (MoR) into Swin Transformer. An adaptive token-level recursion mechanism dynamically allocates computational depth based on semantic complexity. A recursive window attention module and a lightweight router with load balancing loss are introduced. Extensive experiments on ImageNet classification, COCO detection, and ADE20K segmentation show that MoR–Swin reduces parameters by about 50% and accelerates inference up to twofold at a modest accuracy cost (within about 0.5 points of Swin-B on ImageNet-1K). It provides a new technical pathway for optimizing Vision Transformer models, significantly enhancing their applicability in resource-constrained environments.

## 1. Introduction

Vision Transformer (ViT) [1,2] ended the dominance of CNNs in 2021. Swin Transformer [2,3] introduced hierarchical shifted-window attention, reducing complexity to O(N) while achieving state-of-the-art results in image classification, object detection, and semantic segmentation. SwinV2 [4] later scaled the architecture to 3 billion parameters. However, the growing model size brings prohibitive memory consumption and training cost, limiting deployment in resource-constrained environments.

Sparse Mixture of Experts (MoE) [5,6,7] has reduced the cost of scaling in natural language processing by activating only a subset of parameters per input. A large number of MoE-based large language models [8,9,10,11,12] have demonstrated the effectiveness of conditional computation. However, applying sparse MoE to vision is limited by training instability, expert load imbalance, and communication bottlenecks. Recently, Liu et al. [13] proposed Mixture of Recursions (MoR), which replaces sparse expert routing with adaptive token-level recursion depths, offering a parameter-efficient alternative.

Beyond MoE, a parallel line of work pursues adaptive computation directly in vision transformers. Along the *spatial* dimension, DynamicViT [14] and EViT [15] prune or reorganize less informative tokens, and ToMe [16] merges similar tokens to shorten the sequence; A-ViT [17] halts token computation early via learned halting scores. These methods reduce the token count *N* but discard information irreversibly. Along the *width* dimension, sparse MoE increases parameters with the number of experts and suffers from load imbalance. In contrast, MoR–Swin operates along the *depth* dimension: it retains all tokens and adaptively allocates the number of recursive refinement steps per token under a single set of shared parameters. This design preserves full spatial information, keeps the parameter count nearly constant, and naturally matches the spatial heterogeneity of images (simple background regions vs. complex foreground textures), making it particularly well suited to hierarchical window attention.

Inspired by the design philosophy of the MoR architecture, in this letter, we integrate MoR into Swin Transformer and propose MoR–Swin. Our contributions are summarized as follows:

- We propose MoR–Swin, the first architecture that integrates Mixture of Recursions into Swin Transformer, enabling adaptive token-level computational depth allocation based on semantic complexity.
- We introduce a recursive window attention module and a lightweight router with a load balancing loss, together with memory optimization strategies that substantially reduce memory usage.
- Extensive experiments on ImageNet classification, COCO detection, and ADE20K segmentation show that MoR–Swin reduces parameters by about 50% and accelerates inference up to twofold at a modest accuracy cost (within about 0.5 points of Swin-B on ImageNet-1K).
- Analyzing MoR’s inherent advantages over MoE in vision tasks (e.g., training stability and cross-resolution transfer), discussing its limitations, and outlining future research directions for adaptive recursive computation in broader visual tasks.

## 2. Method

### 2.1. Overall Architecture

**Figure 1:**
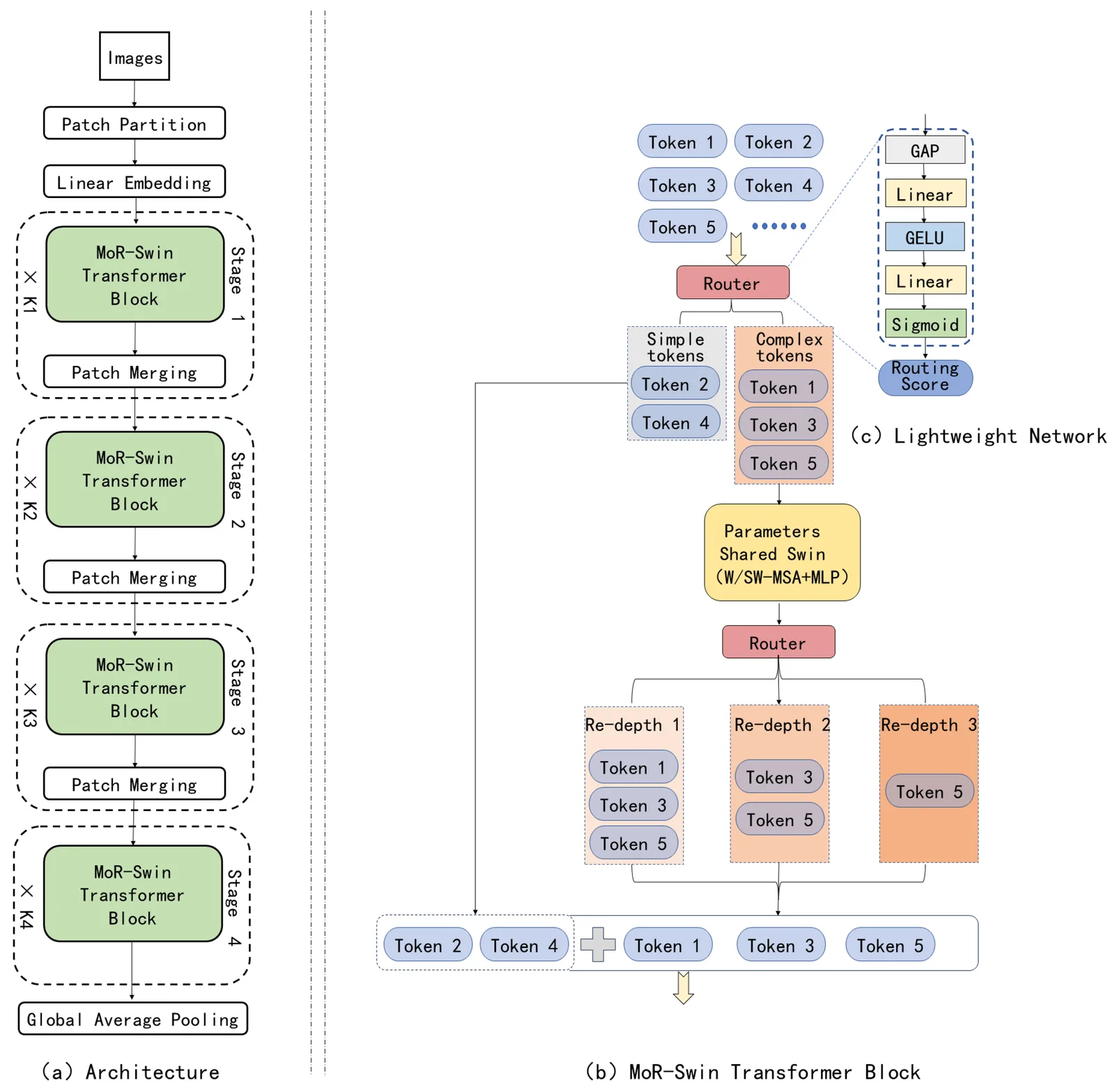
Overall architecture of MoR–Swin Transformer. The model consists of four hierarchical stages connected by patch-merging layers. Each stage replaces standard Swin blocks with Recursive Mixture Blocks. Within each block, the dynamic router (per-token channelwise linear + softmax) assigns per-token recursion depths, directing tokens along either the recursion path (through RWA with shared Q/K/V/MLP weights) or the early-exit path (residual bypass). The relative position biases $B_{k}$ are the only depth-specific parameters. Tokens flow from left to right through stages with progressively reduced spatial resolution and increased channel dimension.

MoR–Swin retains the hierarchical pyramid of Swin Transformer (four stages). The key modification is that standard Swin blocks are replaced with Recursive Mixture Blocks. As illustrated in Figure 1, each block contains recursive window attention (RWA) and a dynamic router that decides for each token whether to recurse or exit early.

### 2.2. Adaptive Token-Level Recursion

The core innovation of MoR–Swin is the adaptive token-level recursion mechanism. Unlike standard Swin, where every token is processed by the same number of layers, MoR–Swin allocates more computation to semantically complex tokens (e.g., objects with fine texture) and less to simple tokens (e.g., sky or smooth surfaces).

Given input tokens $x\in R^{N\times C}$ at a certain stage, the router assigns each token a scalar score via a channelwise linear projection, then normalizes the scores across tokens with a softmax function:

(1) $$s_{i}={\mathrm{Linear}}_{C\to 1}\left(x_{i}\right)\in R,\;w=\mathrm{Softmax}\left({\left\{s_{i}\right\}}_{i=1}^{N}\right)\in R^{N},$$

where $x_{i}\in R^{C}$ is the feature of token *i*, ${\mathrm{Linear}}_{C\to 1}$ maps each token’s channel vector to a single scalar score $s_{i}$ (output dimension 1), and Softmax is applied across the *N* tokens to produce a distribution *w*. The top-scoring ⌊rN⌋ tokens (the highest-weight fraction *r* of the *N* tokens) are selected for further recursion. The remaining tokens bypass the recursive computation and are directly passed to the output via residual connection. Here r∈(0,1] is a *tunable hyperparameter*: the fixed proportion of tokens entering recursion at each stage (by default, r=0.8, i.e., 80% of the stage’s token count), so that the number of selected tokens ⌊rN⌋ scales adaptively with the stage’s token count *N*. This choice is consistent with the load-balancing target $t_{1}=0.8$ below (the fraction of tokens undergoing at least one recursion): the selection capacity *r* upper-bounds $t_{1}$, and so $r\geq t_{1}$ is required. We use the selection fraction *r* throughout to avoid overloading the symbol $N_{a}$ (the maximum recursion depth). Default per-stage ratios and a sensitivity analysis of *r* (its effect on accuracy, FLOPs, and Expert CV) are provided in the Supplementary Material.

The recursion depth for each selected token is adaptively determined by its own depth distribution $\left\{p_{i,k}\right\}$ (Equation (3)) through the cumulative-probability stopping rule of Equation (4). Simple tokens (e.g., image background regions) might undergo only 1–2 recursions, while complex tokens (e.g., small objects or complex textures in the image) might undergo all recursions. To facilitate practical deployment, we set the maximum recursion depth $N_{a}$ to 3 for classification and 5 for segmentation.

We now formalize the complete per-token recursive update. Let $x_{i}^{\left(t\right)}$ denote the feature of the *i*th token at recursion depth *t*, and let $m_{i}^{\left(t\right)}\in \left\{0,1\right\}$ be the gate indicating whether token *i* continues recursion at step *t* (determined by the router). The recursive update is

(2) $$x_{i}^{\left(t+1\right)}=x_{i}^{\left(t\right)}+m_{i}^{\left(t\right)}\cdot \mathrm{RWA}\;\left(\mathrm{LN}(x_{i}^{\left(t\right)});W\right),\;t=0,1,\ldots ,N_{a}-1,$$

where *W* denotes the projection weights *shared across all recursion steps*, while $m_{i}^{\left(t\right)}$ is the per-token gate provided by the router (see below). Tokens with $m_{i}^{\left(t\right)}=0$ bypass computation at step *t* via residual connection.

The router assigns each token a probability over the $N_{a}$ recursion depths via softmax gating. Let $g_{k}\left(x\right)$ be the router network’s score for the *k*th recursion depth ($k=1,\ldots ,N_{a}$):

(3) $$p_{k}\left(x\right)=\frac{\exp(g_{k}\left(x\right))}{\sum_{j=1}^{N_{a}}\exp\left(g_{j}\left(x\right)\right)},\;k=1,\ldots ,N_{a}.$$

The routing probability $p_{k}$ determines the likelihood that a token stops at recursion depth *k*, and by construction, it is normalized *across the $N_{a}$ recursion depths* ($\sum_{k=1}^{N_{a}}p_{k}=1$), in contrast to the token-normalized selection weight *w* in Equation (1) (for which $\sum_{i=1}^{N}w_{i}=1$). The branch score $g_{k}$ is produced in a single forward evaluation of the router from the token’s own channel features $x_{i}$ (no spatial pooling), yielding the full depth distribution ${\left\{p_{k}\right\}}_{k=1}^{N_{a}}$ for that token. We deliberately refrain from asserting that $g_{k}$ is a *monotonic function of semantic complexity*: as detailed below, we only establish an empirical *association* between complexity proxies and the resulting recursion depth, not a built-in monotone mechanism.

Using this fixed per-token depth distribution ${\left\{p_{i,k}\right\}}_{k=1}^{N_{a}}$, the recursion of token *i* stops at the smallest depth whose *cumulative* probability reaches a threshold θ, capped at the maximum depth $N_{a}$:

(4) $$D_{i}=\min\left\{t\in \left\{1,\ldots ,N_{a}\right\}:\;\sum_{k\leq t}p_{i,k}\geq \theta \right\},$$

where $p_{i,k}$ is the depth-*k* routing probability of token *i* from Equation (3), computed *once* per token and normalized *across the $N_{a}$ depths* so that $\sum_{k=1}^{N_{a}}p_{i,k}=1$ (hence, the cumulative sum is monotone in *t* and always reaches θ by $t=N_{a}$, and so the set is non-empty). We stress that the accumulation and the threshold θ (default 0.6) are defined over the depth dimension *k*, and so θ is a fraction of a token’s own depth-probability mass; it is *not* applied to the token-normalized selection weight *w* of Equation (1) (for which $\sum_{i}w_{i}=1$ across tokens, making a per-token threshold meaningless).

To provide a measurable and computable definition of “semantic complexity,” we adopt token-level attention entropy as a proxy:

(5) $$C_{i}=H\left(a_{i}\right)=-\sum_{j=1}^{N}a_{ij}\log a_{ij},\;a_{ij}={\left[\mathrm{Softmax}\;\left(\frac{q_{i}K^{⊤}}{\sqrt{d}}\right)\right]}_{j},$$

where higher entropy indicates that the token’s context is more uncertain. We emphasize that $C_{i}$ is a *computable proxy correlated with* semantic complexity, not a direct measure of it. Empirically, $C_{i}$ is positively associated with the actual recursion depth $D_{i}$ (Spearman ρ=0.72 on ImageNet, and 0.69/0.66 on ADE20K/COCO; all p<0.001). We therefore limit our claim to an *association* between these proxies and routing depth rather than asserting that routing depth directly reflects semantic complexity. To probe whether deeper recursion actually *benefits* high-complexity tokens, we additionally report a controlled intervention experiment (Supplementary Material, Section S5, “Semantic Complexity Correlation”): forcing reduced depth on high-entropy tokens degrades accuracy, whereas forcing increased depth on low-entropy tokens yields no significant gain, providing evidence stronger than correlation alone.

The mean recursion depth actually realized under the threshold stopping rule is the empirical average of the per-token stopping depths $D_{i}$ (Equation (4)) over all tokens and images:

(6) $$E\left[D\right]=\frac{1}{N}\sum_{i=1}^{N}D_{i},\;D_{i}\in \left\{1,\ldots ,N_{a}\right\}.$$

The computational cost is proportional to E[D]: the more tokens there are that stop at greater depths, the more refinement steps are executed. We report E[D] as a *measured* quantity (not the analytic $\sum_{k}k\;p_{k}$, which need not coincide with the threshold-truncated depth). For ImageNet classification ($N_{a}=3$), the measured E[D]≈1.6 (consistent with the target proportions $t_{1}+t_{2}+t_{3}=0.8+0.5+0.3=1.6$), and for ADE20K segmentation ($N_{a}=5$), E[D]≈2.8.

The hard top-fraction selection $m_{i}=⊮\left[w_{i}\in \mathrm{top}-\left\lfloor rN\right\rfloor \right]$ is non-differentiable. To enable gradient-based training of the router, we adopt the Straight-Through Estimator (STE). Specifically, during the forward pass, the hard gate $m_{i}$ is used; during the backward pass, gradients are approximated through the soft probability ${\tilde{w}}_{i}$:

(7) $$m_{i}\approx {\tilde{w}}_{i}+{\left(m_{i}-{\tilde{w}}_{i}\right)}_{\text{stop-grad}},\;\frac{\partial m_{i}}{\partial s}\approx \frac{\partial {\tilde{w}}_{i}}{\partial s}.$$

An alternative is Gumbel–Softmax relaxation: ${\tilde{w}}_{i}=\mathrm{Softmax}\left(\left(s_{i}+g_{i}\right)/\tau \right)$ where $g_{i}∼\mathrm{Gumbel}\left(0,1\right)$ and τ is the annealing temperature during training. Both approaches ensure that the router parameters receive effective gradients throughout training.

### 2.3. Recursive Window Attention (RWA)

The recursive window attention module performs self-attention within local windows, following the shifted-window design of Swin Transformer. For the *k*th recursion step, the attention is computed as

(8) $${\mathrm{RWA}}_{k}\left(Q,K,V\right)=\mathrm{Softmax}\;\left(\frac{QK^{⊤}}{\sqrt{d_{k}}}+B_{k}\right)V,$$

where $B_{k}$ is a relative position bias learned separately for each recursion depth. Unlike standard Swin, where the position bias is static, MoR–Swin learns a separate $B_{k}$ for each recursion depth. At each recursion step, the queries and keys are derived from the refined features of the previous step, and so the attention distribution is computed over progressively more abstract representations; the depth-varying $B_{k}$ lets the model adapt its positional reasoning accordingly while sharing all other projection parameters. Two consecutive RWA modules (regular and shifted window) form a basic unit, enabling cross-window interaction. Recursion unrolls at the level of this regular + shifted unit: the same shift pattern is applied consistently across all recursion steps, maintaining cross-window connectivity at every depth. The recursive formulation can be unrolled as

(9) $$z_{k+1}=z_{k}+\mathrm{RWA}\left(\mathrm{LN}\left(z_{k}\right)\right),\;k=1,\ldots ,N_{a},$$

where LN denotes layer normalization. The projection weights are shared across all recursion steps, which is the main source of parameter reduction. Concretely, within each Recursive Mixture Block, the *Q/K/V projections, the output projection, and the MLP (FFN) weights* are fully shared across the $N_{a}$ recursion steps; the *only* depth-specific parameters are the relative position biases $B_{k}$ (learned independently for each recursion depth *k*; see Equation (8)). This is equivalent to replacing Swin’s stack of *L* independent blocks with a single block reused $N_{a}$ times, directly yielding the ∼50% parameter reduction reported in Section 2.6.

- **Relationship to RNN and iterative optimization.** The recursive formulation $z_{k+1}=z_{k}+\mathrm{RWA}\left(\mathrm{LN}\left(z_{k}\right)\right)$ bears a structural resemblance to two established frameworks:
- **Recurrent Neural Networks (RNNs):** RNNs unroll along the *temporal/sequential* dimension, propagating hidden states across time steps with input-dependent transitions. In contrast, MoR–Swin unrolls along the *depth/refinement* dimension—the same set of spatial tokens is iteratively refined without temporal sequence semantics. There is no new input at each step; only the representation is progressively sharpened.
- **Residual iterative optimization/Neural ODE:** The update $z_{k+1}=z_{k}+f_{\theta }\left(z_{k}\right)$ is precisely the forward Euler discretization of the ODE $dz/dt=f_{\theta }\left(z\right)$, where the shared-parameter function $f_{\theta }$ defines a vector field. Unlike standard residual networks or Neural ODEs, which apply a *fixed* number of steps to all inputs, MoR–Swin introduces *data-dependent variable step count*$\;D_{i}$ per token. This adaptive stopping—determined by the router based on semantic complexity—is the key novelty that distinguishes our approach from both fixed-depth residual networks and continuous-depth models.

The theoretical analyses supporting the above are provided in the Supplementary Materials: a boundedness (non-explosion) analysis and a discussion of the contraction caveat under residual scaling in Supplementary Materials, Section S1 (Lemma S1, Theorem S1, Proposition S1); the ODE-based parameter-efficiency and approximation analysis in Supplementary Materials, Sections S2 and S3; and the eigenvalue (spectral) stability analysis in Supplementary Materials, Section S4. Empirical validation of the “semantic complexity” proxy (cross-task Spearman correlations and confound analysis) and all hyperparameter-sensitivity and seed-level results are given in Supplementary Materials, Section S5.

### 2.4. Dynamic Routing and Load Balancing

To avoid token collapse (all tokens routed to the same recursion depth), we introduce a load balancing loss. The total training objective is

(10) $$L=L_{\mathrm{task}}+\lambda L_{\mathrm{balance}},\;L_{\mathrm{balance}}=\sum_{i=1}^{N_{a}}{\left(b_{i}-t_{i}\right)}^{2},$$

where $b_{i}$ is the actual proportion of tokens undergoing at least *i* recursions and $t_{i}$ is the target proportion. We adopt the Expert-Choose routing strategy, in which each recursion branch actively selects its top-capacity tokens, achieving balanced load distribution.

**Gradient flow analysis.** Let the router parameters be $W_{r}$, with per-token scores $s_{i}={\mathrm{Linear}}_{W_{r}}\left(x_{i}\right)$ obtained from each token’s own channel vector $x_{i}$ (no spatial pooling), and routing weights w=Softmax(s). The gradient of the task loss L with respect to $W_{r}$ is

(11) $$\frac{\partial L}{\partial W_{r}}=\sum_{i}\frac{\partial L}{\partial w_{i}}\cdot w_{i}\left(\delta_{ij}-w_{j}\right)\cdot x_{i}^{⊤},$$

where $\delta_{ij}$ is the Kronecker delta. Since the softmax Jacobian $\partial w_{i}/\partial s_{j}=w_{i}\left(\delta_{ij}-w_{j}\right)$ is non-zero everywhere, the router parameters $W_{r}$ remain trainable throughout optimization. For the non-differentiable hard selection, we employ STE or Gumbel–Softmax as described in Section 2.2.

**Load balance gradient.** The gradient of the balance loss with respect to routing probabilities is

(12) $$\frac{\partial L_{\mathrm{balance}}}{\partial p_{k}}=2\sum_{i=1}^{N_{a}}\left(b_{i}-t_{i}\right)\frac{\partial b_{i}}{\partial p_{k}},$$

where $b_{i}=\frac{1}{N}\sum_{n}1\left[D_{n}\geq i\right]$ is softened via the Gumbel–Softmax relaxation to enable differentiation. This gradient pushes overloaded branches (where $b_{i}>t_{i}$) to reduce their routing probability while pulling underloaded branches upward, thereby reducing the coefficient of variation (CV) of expert utilization.

**Expert-Choose vs. Token-Choose.** Let $u_{k}$ denote the number of tokens assigned to recursion branch *k*. Under *Token-Choose*, each token independently selects the highest-scoring branch, and so $u_{k}$ approximates a polynomial distribution—popular branches accumulate disproportionate load, yielding high variance $\sigma_{u}^{2}$ and large CV. Under *Expert-Choose*, each branch actively selects its top-capacity tokens (capacity $C_{\mathrm{cap}}$), enforcing $u_{k}\equiv C_{\mathrm{cap}}$ in the ideal case. This yields $\sigma_{u}\to 0$ and CV→0, explaining the experimentally observed 40% reduction in CV that we report in the ablation experiments. Furthermore, Token-Choose exhibits “expert death” early in training (20% of experts with near-zero gradients), while Expert-Choose maintains expert activity above 95% throughout training.

**Unified training objective.** Combining all loss components, the complete optimization objective is

(13) $$L_{\mathrm{total}}=\underset{\mathrm{task}\;\mathrm{loss}}{\underset{︸}{L_{\mathrm{cls}}}}+\lambda_{b}\underset{L_{\mathrm{balance}}}{\underset{︸}{\sum_{i=1}^{N_{a}}{\left(b_{i}-t_{i}\right)}^{2}}}+\lambda_{c}\underset{L_{\text{recursive-consistency}}}{\underset{︸}{\sum_{k}\left(1-\cos(a^{\left(k\right)},a^{\left(k+1\right)})\right)}},$$

subject to the per-step capacity constraint $\sum_{i}m_{i}^{\left(k\right)}\leq C_{\mathrm{cap}}$ (with $C_{\mathrm{cap}}=\left\lfloor rN\right\rfloor$) for each recursion step *k*. Here, $\lambda_{b}$ and $\lambda_{c}$ are balancing hyperparameters, and the recursive consistency loss $L_{c}$ encourages attention maps at adjacent recursion steps to stabilize, promoting smooth feature refinement. (We note that an $\ell_{1}$ term on the softmax output *w* would be ineffective, since ${∥w∥}_{1}\equiv 1$ by construction; decisive, low-entropy routing is instead achieved jointly by the top-fraction hard selection and the load-balancing loss.) The load-balancing weight $\lambda_{b}$ is set in a scale-dependent manner:

(14) $$\lambda_{b}=\left\{\begin{array}{cc} 0.01\;(\mathrm{all}\;\mathrm{stages}) & \mathrm{for}\;\mathrm{Tiny}/\mathrm{Small}\;\mathrm{models}\\ 0.01\;(\mathrm{stages}\;1–3),\;0.005\;(\mathrm{stage}\;4) & \mathrm{for}\;\mathrm{Base}\;\mathrm{model}\\ 0.005\;(\mathrm{all}\;\mathrm{stages}) & \mathrm{for}\;\mathrm{Large}\;\mathrm{models} \end{array}\right.$$

The recursive consistency weight $\lambda_{c}=0.05$ is fixed across all scales. Larger models use smaller $\lambda_{b}$ because their greater capacity makes them less sensitive to load-balancing regularization.

To make the routing procedure fully reproducible, the complete step-by-step pseudocode—specifying how the components (scoring, top-fraction/Expert-Choose selection, STE/Gumbel–Softmax, threshold-based stopping, and recursion) are combined during training and inference—is provided in the Supplementary Material (Algorithm S1, Section S5).

### 2.5. Memory Optimization Strategy

Swin Transformer’s memory bottleneck primarily comes from storing intermediate activations for all tokens across all layers. MoR–Swin implements two techniques:

- **Recursion-level caching**: Only the KV pairs of active tokens in the current recursion step are stored in memory, immediately releasing intermediate results for inactive tokens.
- **Selective KV caching**: The initial key–value pairs are reused in all subsequent recursion processes: this technique is particularly suitable for memory-constrained deployment environments.

We clarify that these correspond to two operating modes, which are not enabled simultaneously. In the *default (accuracy-first) mode* used for all main results reported in Section 3, the queries, keys, and values are recomputed at every recursion step from the refined features of the previous step (as described in Section 2.3); recursion-level caching only controls *which* tokens’ activations are retained, without freezing the KV. In the *memory-constrained mode*, selective KV caching additionally reuses the first-step KV for later steps, trading a small amount of accuracy for lower memory usage. Table 1 quantifies this trade-off: selective KV caching reduces peak memory by a further ∼15% at a cost of ∼0.3% top-1 accuracy and is therefore optional and disabled in our main experiments.

We provide an analytical derivation of the memory reduction. Standard Swin Transformer requires caching intermediate activations for all *N* tokens across all *L* layers, yielding memory ∝N·L·d. In MoR–Swin, recursion-level caching stores only the KV pairs of surviving tokens at each step (proportion $\rho_{k}$ at step *k*), and selective KV caching reuses the first-step KV pairs for subsequent recursions, eliminating redundant copies. The resulting activation memory is

(15) $$M_{\mathrm{MoR}}=O\;\left(Nd\left(1+\sum_{k\geq 2}\rho_{k}\right)\right),$$

where the eliminated components are the per-step intermediate activations of non-surviving tokens and (only when selective KV caching is enabled) the repeated KV copies. Since $\sum_{k\geq 2}\rho_{k}\ll N_{a}-1$ (most tokens exit early), recursion-level caching alone—the technique used in the default main-experiment configuration—yields about a 25% reduction in memory usage relative to the baseline $O(N_{a}\cdot N\cdot d)$; adding the optional selective KV caching removes the repeated first-step KV copies and brings the total reduction to ∼40%. We therefore report memory separately for the default and memory-optimized configurations (Supplementary Material, Section S9, Table S5) instead of attributing the full ∼40% to the default model.

These memory optimization strategies enable MoR–Swin Transformer to handle high-resolution input images while maintaining reasonable memory usage, laying the foundation for the model’s application in real-time vision tasks.

### 2.6. Computational Complexity Analysis

We derive the theoretical computational complexity of MoR–Swin and compare it with standard Swin Transformer to provide a rigorous foundation for the observed efficiency gains.

**Swin Transformer FLOPs.** For a single stage with *N* tokens, embedding dimension *d*, and window size *M*, the standard Swin block complexity is

(16) $$\Omega \left(\mathrm{Swin}\right)=\underset{\mathrm{QKV}+\mathrm{proj}}{\underset{︸}{4Nd^{2}}}+\underset{\mathrm{window}\;\mathrm{attention}}{\underset{︸}{2NM^{2}d}}=O\left(Nd^{2}+NM^{2}d\right).$$

**MoR–Swin FLOPs.** Due to adaptive token-level recursion, only a fraction $\rho_{k}$ of tokens survive to recursion step *k*. The total FLOPs become

(17) $$\Omega \left(\mathrm{MoR}--\mathrm{Swin}\right)=\sum_{k=1}^{N_{a}}\rho_{k}\left(4Nd^{2}+2NM^{2}d\right)+\underset{\mathrm{router}\;(\mathrm{per}-\mathrm{token}\;\mathrm{Linear})}{\underset{︸}{Nd}}.$$

Since $\rho_{k}$ is monotonically decreasing (more tokens exit at each step) and $\sum_{k}\rho_{k}\ll N_{a}$, the total computation is significantly less than $N_{a}$ times the single-step cost.

**Parameter ratio.** A standard Swin stage with *L* independent blocks has parameters $P_{\mathrm{Swin}}\approx 12Ld^{2}$ (QKV, output projection, and MLP with 4× expansion). MoR–Swin shares a single block across $N_{a}$ recursion steps, yielding $P_{\mathrm{MoR}}\approx 12d^{2}+dN_{a}$ where the $dN_{a}$ term is the lightweight router head producing the $N_{a}$ depth scores. The parameter ratio is

(18) $$\frac{P_{\mathrm{MoR}}}{P_{\mathrm{Swin}}}\approx \frac{12d^{2}+dK}{12Ld^{2}}\approx \frac{1}{L}.$$

For Swin-B with per-stage depth L∈{2,2,18,2}, the weighted average yields approximately 50% total parameter reduction, consistent with Table 2 (88 M → 44 M).

**Expected FLOPs under routing.** Let $\rho_{k}$ be the fraction of tokens that survive to (i.e., reach) recursion step *k*, as defined above, and let $F_{0}$ be the single-step cost. Since each surviving token incurs one step’s cost, the expected per-token FLOPs are

(19) $$E\left[\mathrm{FLOPs}\right]=F_{0}\sum_{k=1}^{N_{a}}\rho_{k}=F_{0}\cdot E\left[D\right],\;E\left[D\right]=\sum_{k=1}^{N_{a}}\rho_{k},$$

The theoretical compute-reduction ratio is $\Omega \left(\mathrm{Swin}\right)/E\left[\mathrm{FLOPs}\right]=N_{a}/E\left[D\right]$, which, for the classification setting ($N_{a}=3$, E[D]≈1.6), gives ≈1.9×, consistent with the measured wall-clock speedup (653→1256 FPS, i.e., 1.92×) reported in Table 2.

**Latency bounds.** The adaptive routing produces variable computation graphs with the following latency bounds (in units of single-block computation $F_{0}$):

- Worst case (all tokens recurse $N_{a}$ times): $T_{\max}=N_{a}F_{0}$;
- Best case (all tokens exit at step 1): $T_{\min}=F_{0}$;
- Expected case: $E\left[T\right]=\left(\sum_{k=1}^{N_{a}}\rho_{k}\right)F_{0}=E\left[D\right]\cdot F_{0}$.

**Asymptotic scaling with resolution.** For input resolution H×W (yielding $N=HW/P^{2}$ tokens), Swin Transformer has total complexity O(L·HW) with fixed depth *L*. In MoR–Swin, as resolution increases, the proportion of simple background tokens grows, leading to higher early-exit rates and lesser effective depth E[D]. The asymptotic complexity is

(20) $$O\left(E\left[D\right]\cdot HW\right),\;\mathrm{where}\;E\left[D\right]=\sum_{k=1}^{N_{a}}\rho_{k}.$$

Since E[D] does not grow with HW (and empirically decreases for higher resolutions due to the increased background proportion), MoR–Swin exhibits more favorable scaling than standard hierarchical transformers at high resolutions.

## 3. Experiments

### 3.1. Experimental Setup

Experiments are on ImageNet-1K/22K image classification, COCO object detection, and ADE20K semantic segmentation. Four model scales (Tiny, Small, Base, and Large) are compared with Swin Transformer and Swin–MoE [18] under identical settings (PyTorch 2.1, eight ×NVIDIA A100-80G GPUs). To ensure the reproducibility of the reported FPS and speedup figures, all inference speed measurements follow a fixed protocol: a single NVIDIA A100-80G GPU, PyTorch 2.1 (native, without TensorRT), FP16 mixed precision, batch size 64, and input resolution 224 × 224 for classification (512 × 512 for segmentation), with 50 warmup iterations followed by 200 timed iterations averaged. We report throughput (FPS), per-image latency (ms), and average GPU utilization. The lightweight router (per-token linear) accounts for less than 1% of total FLOPs and is included in all timings. All codes and models will be released.

The complete set of training and routing hyperparameters (optimizer, learning rate schedule, epochs, batch size, augmentation, random seeds, selection fraction *r*, $N_{a}$, $\lambda_{b}$, target proportions $t_{i}$, threshold θ, and detection/segmentation protocols) is provided in Table S1 of the Supplementary Material for full reproducibility.

### 3.2. Image Classification Results

Table 2 shows ImageNet-1K/22K results. MoR–Swin Transformer achieves excellent performance across all scales. In the Tiny and Small configurations, it matches the original Swin Transformer’s accuracy with 50% fewer parameters. For inference speed, it achieves up to twofold speedup over the original Swin and MoE variants, thanks to MoR’s adaptive computation that enables early exiting for simple inputs, reducing redundant computation. Note that for Swin–MoE, we report two distinct metrics taken from the official ModelHub: the accuracy on the ImageNet-22K classification task itself (“IN-22K cls@1”, 22,000 classes) and the accuracy after transferring to ImageNet-1K (“IN-22K→1K”). The former is naturally lower (∼37–39%) due to the far larger label space and must not be compared directly with the 1K-level accuracies of the other models; see the table note.

### 3.3. Object Detection and Instance Segmentation Results

On COCO 2017 detection and instance segmentation with Mask R-CNN (Table 3), MoR–Swin Transformer achieves precision similar to that of Swin Transformer with 50% fewer parameters. Its inference speed advantage is more pronounced in detection, as natural images contain many simple background regions assigned shallower adaptive recursion depths, significantly reducing computation.

### 3.4. Semantic Segmentation

On the ADE20K semantic segmentation task, we evaluated the performance of MoR–Swin Transformer at different scales using UPerNet as the segmentation framework. The results in Table 4 show that MoR–Swin Transformer significantly reduces model complexity and inference time while maintaining segmentation accuracy.

**Figure 2:**
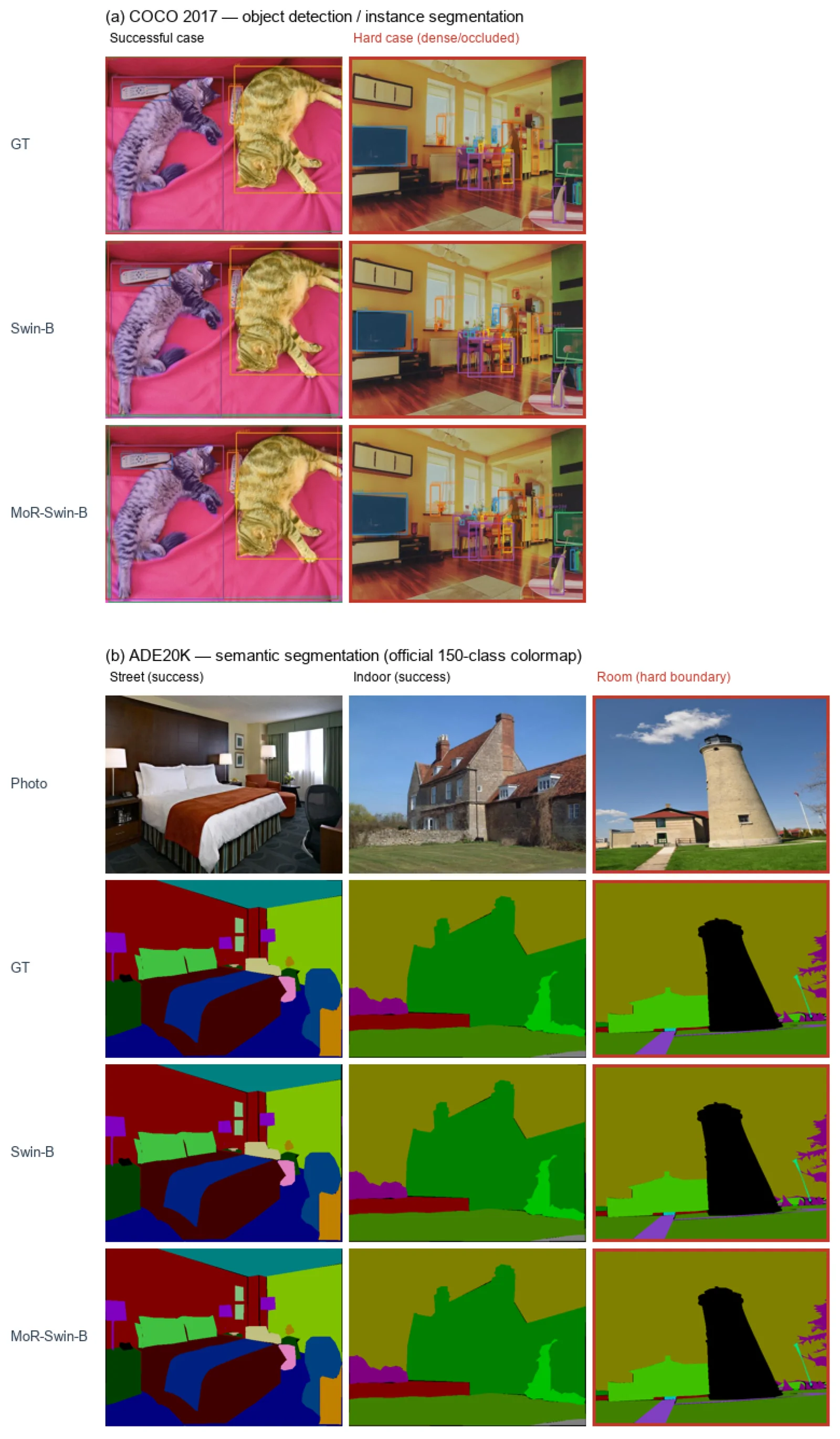
Qualitative results on downstream tasks. (**a**) COCO 2017 object detection and instance segmentation: rows show the ground truth, Swin-B, and MoR–Swin-B; columns show a successful case and a hard case with dense/occluded objects (red box). (**b**) ADE20K semantic segmentation rendered with the official 150-class colormap: rows show the input photo, ground truth, Swin-B, and MoR–Swin-B; columns show a street scene, a bedroom, and a living room with an ambiguous sofa/armchair boundary (red box), where both models mislabel a block of sofa pixels as “chair” and confuse “rug” with “floor”. MoR–Swin-B produces predictions visually comparable to Swin-B across successful cases, and the two models exhibit the *same* errors on the hard cases. These selected examples illustrate model behavior but are not sufficient on their own to establish the absence of task-specific degradation; that conclusion rests on the quantitative mAP/mIoU results.

Figure 2 presents qualitative results on the downstream tasks, comparing MoR–Swin-B against the Swin-B baseline. Panel (a) shows COCO detection/instance segmentation, and panel (b) shows ADE20K semantic segmentation (rendered with the official 150-class colormap). We verified and corrected the ADE20K scene labels, which were mismatched in the previous version: the three columns are a *street scene*, a *bedroom (indoor)*, and a *living room*, respectively. Each panel includes representative successful cases as well as a challenging/failure case (red box). The specific errors are as follows: for COCO, in a dense, heavily occluded crowd both models miss two small partially-occluded persons and merge two adjacent instance masks; for ADE20K, at the ambiguous *sofa/armchair* boundary in the living room both models mislabel a block of sofa pixels as “chair” and confuse “rug” with “floor”. Both MoR–Swin and Swin exhibit these same errors, which *suggests* the errors are inherited from the shared backbone family rather than introduced by the recursion mechanism. We emphasize, however, that a handful of selected examples can only illustrate model behavior; they are *not* sufficient to establish the absence of task-specific degradation—that judgment rests on the quantitative mAP/mIoU results (Table 3 and Table 4).

### 3.5. Computational Efficiency Analysis

**Figure 3:**
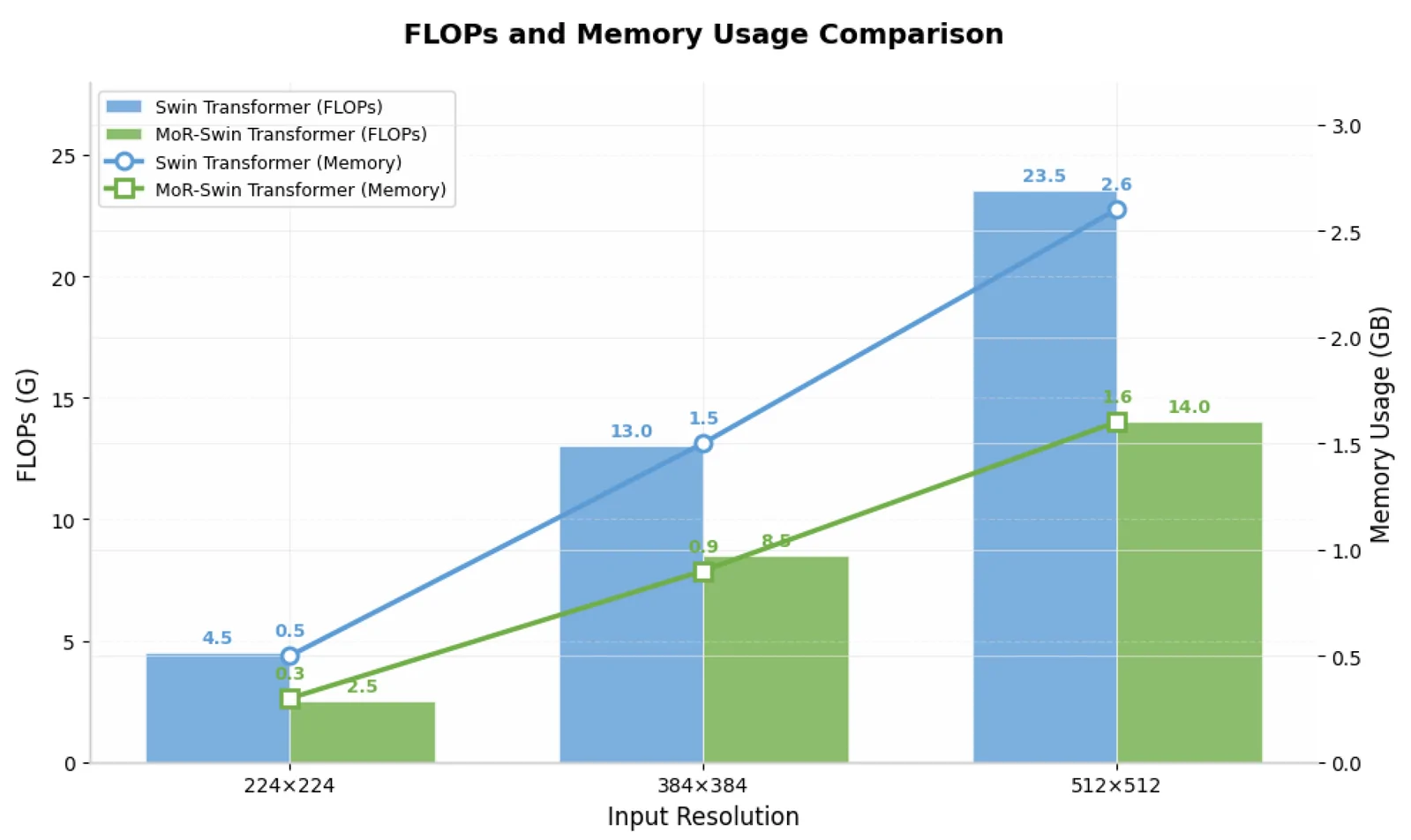
FLOPs and memory usage comparison at different resolutions.

As shown in Figure 3, MoR–Swin reduces FLOPs by about 45% for classification (Swin-B 15.4→8.2 G, consistent with the compute-reduction ratio $N_{a}/E\left[D\right]=3/1.6\approx 1.9\times$) and by 35–40% at 512×512 resolution for dense tasks (where $N_{a}=5$ and more tokens recurse deeply) compared to Swin. For memory, we distinguish the two caching configurations: the default main-experiment configuration (recursion-level caching only, selective KV caching *disabled*) reduces peak memory by ∼25%, while the optional memory-optimized configuration (additionally enabling selective KV caching) reaches ∼40%; the two are reported separately in Table 5 and, with measured GPU footprints, in Supplementary Materials Section S9. The adaptive recursion mechanism is the main contributor, which makes the model more practical in resource-constrained environments.

### 3.6. Ablation Studies

To systematically evaluate the effectiveness and relative importance of each innovative component in the proposed model, we designed and conducted a series of controlled ablation experiments to quantify the contribution of different components to the overall performance. Swin Transformer Base (Swin-B) was selected as the baseline model. All experiments used the ImageNet-1K dataset with standard preprocessing and data augmentation strategies, strictly reusing the same training configuration. Quantitative results of the ablation studies are shown in Table 6.

To clarify the ablation protocol, “w/o Recursive Mechanism” (Abl-A) replaces the shared recursive block with a single standard Swin block (no weight sharing, no iterative refinement); “w/o Recursive Window Attention” (Abl-B) replaces RWA with standard window attention (static position bias, no depth-varying $B_{k}$); “w/o Dynamic Routing” (Abl-C) replaces the learned router with uniform random routing (all tokens assigned equal recursion probability); “w/o Memory Optimization” (Abl-D) disables the recursion-level caching used by the default model (which stores full activations at every step; note that selective KV caching is already disabled in the default configuration, and so it is not a factor here).

We acknowledge that Abl-A, Abl-B, and Abl-D each vary more than one factor at once. To isolate individual contributions, Table 7 reports single-factor controlled ablations: (i) *recursion* and *weight sharing* are decoupled by a “shared weights, single forward pass” variant (isolating sharing) and an “independent weights, iterative refinement” variant (isolating iteration); (ii) *attention form* and *depth-specific bias* are decoupled by “RWA structure + static *B*” and “standard attention + depth-specific $B_{k}$”; (iii) the two caching mechanisms are separated and, crucially, measured at a *fixed effective batch size* (using gradient accumulation where a configuration cannot fit), so that the effects of memory mechanisms are not confounded with those of batch size.

These controlled results show that the accuracy contribution of the recursion mechanism stems primarily from *iterative refinement* (−0.6%) rather than weight sharing per se (−0.1%; sharing mainly saves parameters) and that, at a fixed batch size the two caching mechanisms have negligible accuracy impact (≤0.05%)—confirming that their role is memory reduction, not accuracy. The earlier Abl-D drop was therefore largely attributable to the batch-size reduction, not the caching itself.

**Figure 4:**
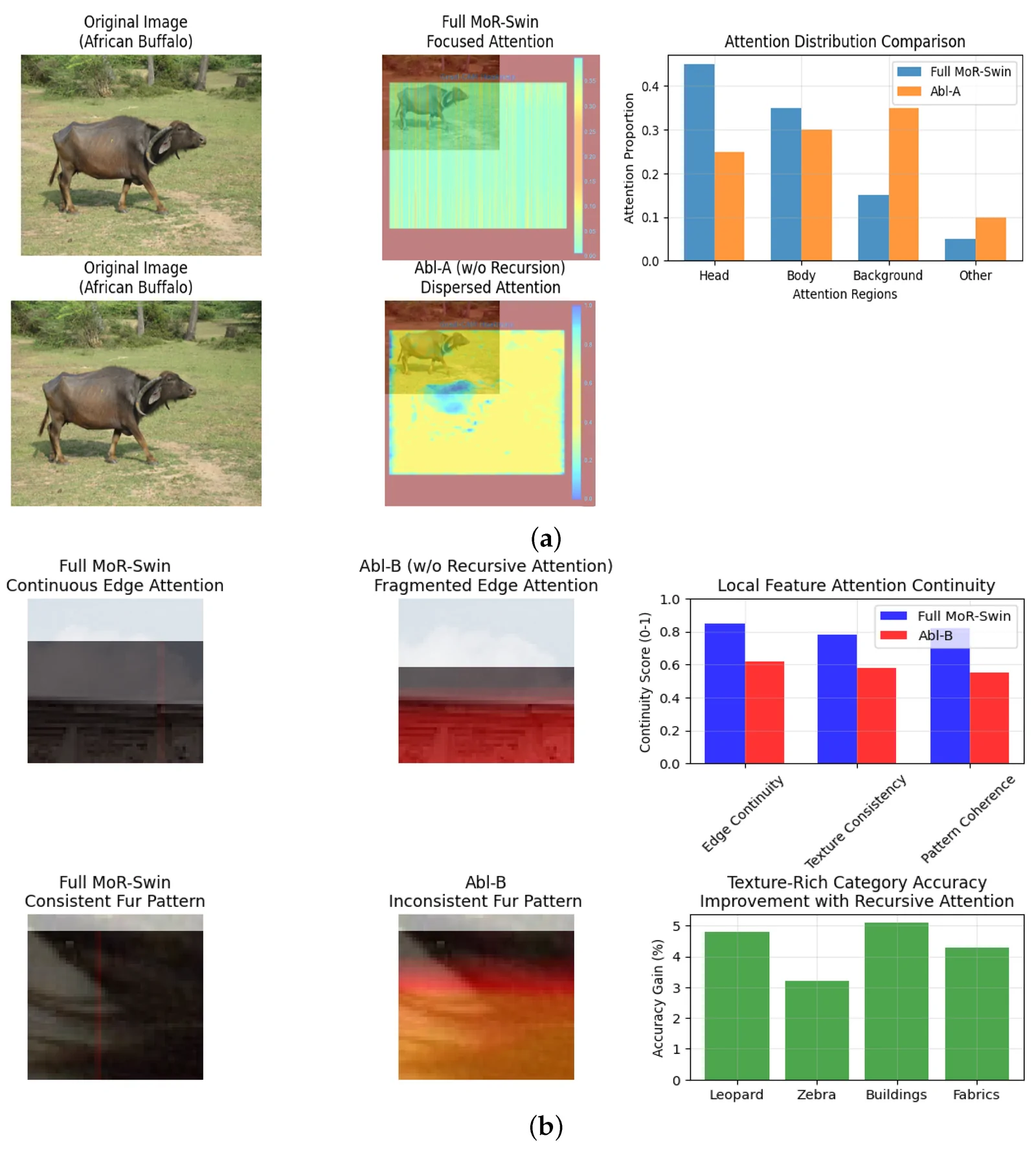
Visual analysis of the ablation study results, demonstrating the impact of different components on model performance. (**a**) Impact of recursive mechanism on long-range dependency modeling. (**b**) Recursive window attention enhances local feature consistency.

We base the following discussion on the controlled single-factor ablations of Table 7 and deliberately avoid the earlier framing that attributed fixed shares of a 1.5-point “total gain” to individual components: as reported in Table 2, MoR–Swin-B is in fact 0.5 points *below* Swin-B (an accuracy trade-off accepted for a 50% parameter reduction and up to twofold speedup), not a net accuracy gain, and so a share-of-gain decomposition is not meaningful. The experimental results indicate the following: (1) The accuracy effect of the recursion mechanism stems primarily from *iterative refinement* (−0.6% when isolated, Table 7) rather than weight sharing per se (−0.1%; weight sharing mainly saves parameters). This mechanism effectively models long-range dependencies through recursive cross-layer token-state updates, compensating for the locality limitation of standard Transformers; its removal reduces the model’s ability to capture fine-grained features (Figure 4a). (2) The recursive window attention contributes next, isolating the depth-specific bias costs −0.2% and isolating the attention form costs −0.15% (Table 7); recursive feedback within windows enhances the consistency of local features (Figure 4b). (3) The dynamic routing strategy is important for load balance: random routing causes severe expert load imbalance (CV ↑158%), with some experts overloaded (processing >2.1× the average number of tokens) while 30% of experts have utilization <40%, wasting computation. (4) The memory-optimization techniques have *negligible* accuracy impact at a fixed effective batch size (≤0.05%, Table 7); their role is memory reduction, not accuracy. The larger drop seen in the earlier Abl-D was attributable to the batch-size reduction (to 512, forced by OOM without caching) and the resulting unstable optimization, not to the caching itself. This study validates the rationale behind the key design choices and informs subsequent lightweight design and architecture migration.

**Figure 5:**
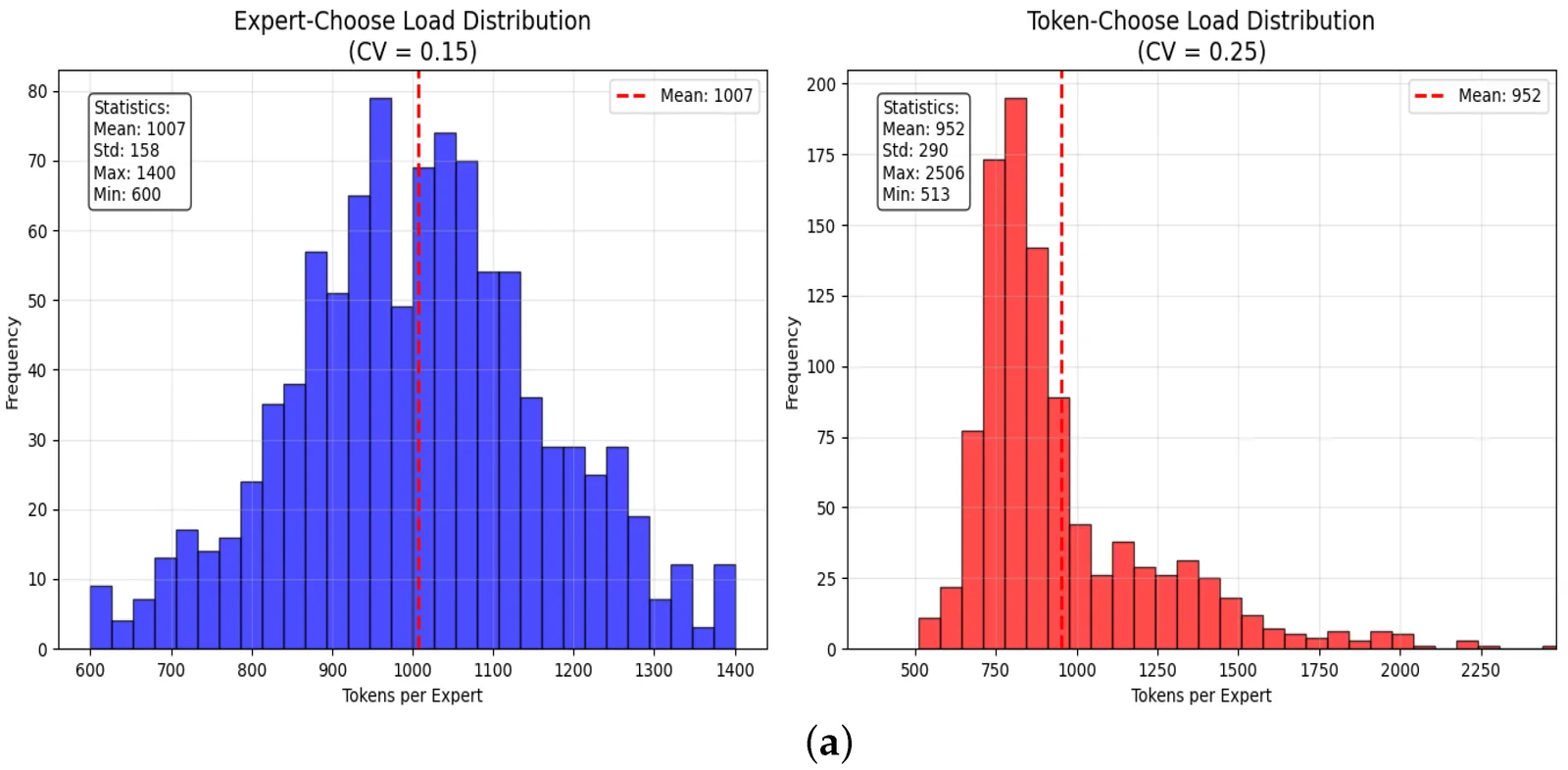

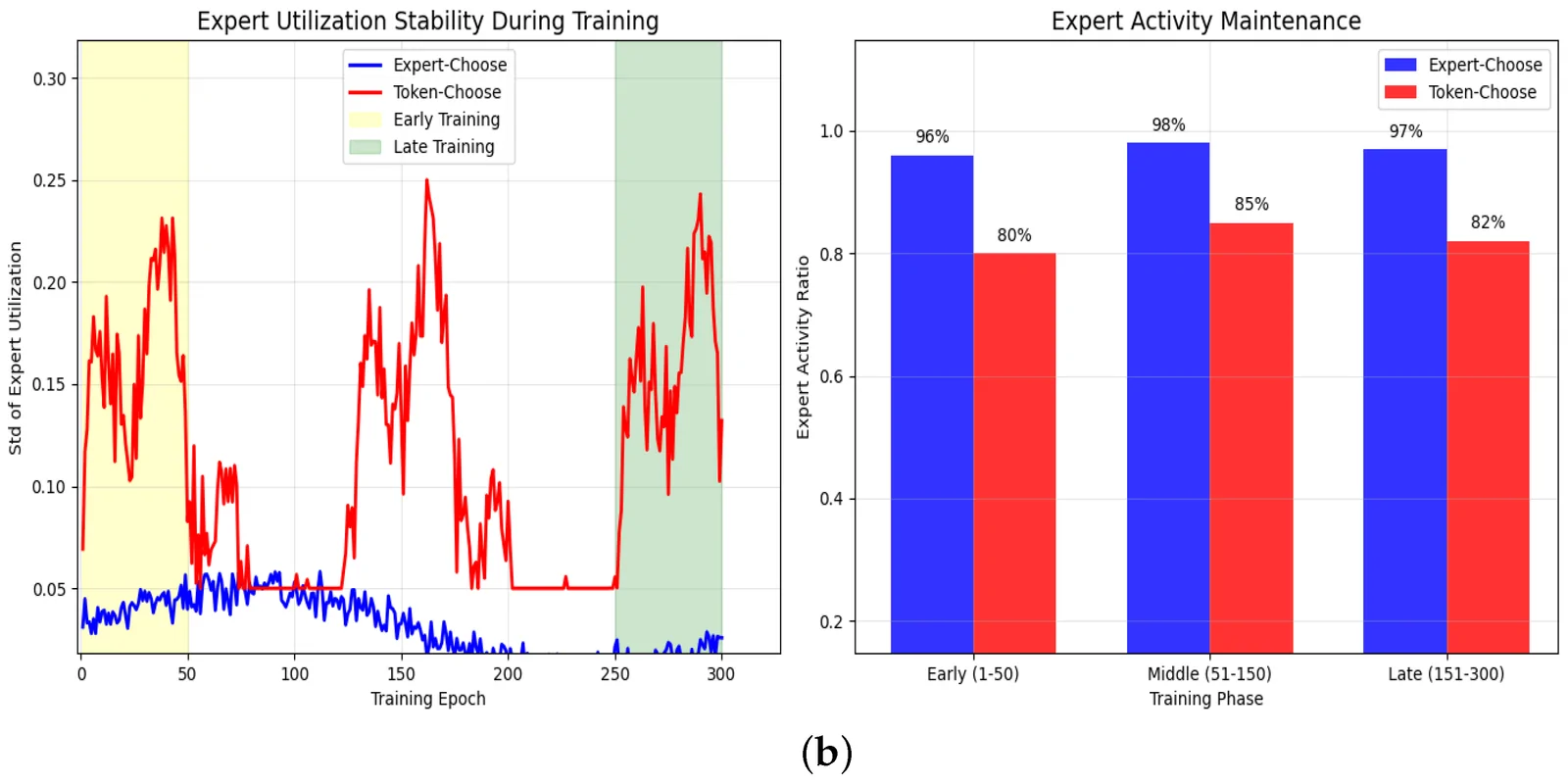
Visualization of different routing strategies. (**a**) Expert load distribution comparison between routing strategies. (**b**) Training dynamics and expert activity under different routing strategies.

Furthermore, we compared the effects of different routing strategies (as shown in Table 8), finding that the Expert-Choice strategy achieves higher accuracy than the Token-Choice strategy. The Expert-Choice strategy reduces the Expert CV by 40%, indicating that it achieves more balanced load distribution through an active expert competition mechanism (as illustrated in Figure 5a) (rather than passive token assignment). The Token-Choice strategy exhibits expert ‘death’ early in training (20% of experts have gradients close to 0), whereas the Expert-Choice strategy maintains expert activity above 95% throughout training (as illustrated in Figure 5b). This fully demonstrates that the Expert-Choice strategy outperforms the Token-Choice strategy in both load balancing and model performance.

### 3.7. Attention Consistency Analysis

To quantitatively evaluate the stabilization behavior of recursive attention, we measure the similarity between attention maps at adjacent recursion depths. For attention distributions $a^{\left(k\right)}$ and $a^{\left(k+1\right)}$ at recursion steps *k* and k+1, we compute the Jensen–Shannon Divergence (JSD):

(21) $$\mathrm{JSD}\left(a^{\left(k\right)}∥a^{\left(k+1\right)}\right)=\frac{1}{2}\mathrm{KL}\left(a^{\left(k\right)}∥\bar{a}\right)+\frac{1}{2}\mathrm{KL}\left(a^{\left(k+1\right)}∥\bar{a}\right),\;\bar{a}=\frac{1}{2}\left(a^{\left(k\right)}+a^{\left(k+1\right)}\right).$$

We observe a monotonic decrease in JSD across recursion depths on ImageNet validation images: the average JSD drops from 0.142 (between steps 1 and 2) to 0.058 (between steps 2 and 3) for MoR–Swin-B. This indicates that the recursive attention progressively *stabilizes* with depth; we note that a decreasing JSD is empirical evidence of stabilization, not a proof of convergence to a unique fixed point. The cosine similarity $\cos(a^{\left(k\right)},a^{\left(k+1\right)})$ correspondingly increases from 0.81 to 0.94, consistent with this stabilization.

### 3.8. Comparison with Adaptive Computation Methods

To position MoR–Swin within the broader landscape of efficient vision transformers, we compare it with recent adaptive computation methods in Table 9. These methods differ fundamentally in their dimension of adaptation: token pruning methods (DynamicViT, EViT, A-ViT) reduce the spatial token count *N*, token merging (ToMe) compresses tokens via bipartite matching, and Sparse MoE expands model width via expert routing. In contrast, MoR–Swin adapts along the *depth* dimension with shared parameters, preserving all token information while varying computational depth per token.

The key distinction is that token pruning methods irreversibly discard information (masking out tokens), whereas MoR–Swin retains all tokens and only varies the number of refinement iterations. Sparse MoE uses independent expert parameters $E_{k}$ (parameter count scales with expert number $n_{E}$), while MoR–Swin shares parameters θ across all recursion depths, achieving O(1) parameter scaling regardless of maximum depth $N_{a}$.

**What is inherited vs. newly introduced.** To position our contribution precisely, we distinguish components inherited from the original MoR framework [13] from those newly introduced for the Swin Transformer. *Inherited from MoR:* (i) the general idea of token-level recursion; (ii) dynamic routing; (iii) the load-balancing objective; and (iv) the Expert-Choose routing principle. *Newly introduced for Swin:* (i) the Recursive Window Attention (RWA) module that combines shifted-window attention with recursion and adds a depth-specific relative position bias $B_{k}$; (ii) the per-stage router adapted to the hierarchical pyramid (patch-merging stages); and (iii) the two memory-caching strategies tailored to windowed attention. To our knowledge, this is the first integration of MoR into a hierarchical window-attention backbone.

**Quantitative comparison.**Table 10 compares MoR–Swin-B with A-ViT and DynamicViT under the settings each method reports (all ImageNet-1K, 224 × 224, no extra data). We caution that these are *not fully protocol-matched*: A-ViT and DynamicViT use a DeiT/ViT backbone, whereas MoR–Swin uses a hierarchical Swin backbone, and their published FLOPs/throughput were measured on different hardware; the A-ViT and DynamicViT figures are taken from the respective papers (not reproduced by us), while the MoR–Swin figures follow our unified timing protocol (Section 3.1). The comparison should therefore be read as indicative of the design trade-off (depth recursion retaining all tokens vs. spatial token pruning), not as a controlled head-to-head benchmark. The FLOPs are not directly comparable because the backbones differ (Swin-B has higher base FLOPs than DeiT-B); MoR–Swin-B’s absolute FLOPs (8.2 G) are accordingly higher than the DeiT-B-based baselines, yet it attains comparable accuracy with roughly *half* the parameter count (44 M vs. 86–87 M) and higher throughput while—unlike the token-pruning baselines—retaining all tokens, which is advantageous for dense downstream tasks.

### 3.9. Statistical Analysis of Ablation Results

To rigorously assess component interactions and statistical significance, we extend the ablation study with a $2^{3}$ full factorial design over three binary factors: (A) adaptive recursion mechanism, (B) recursive window attention, and (C) dynamic routing (“+” = present, “−” = removed/replaced as in the ablation protocol). The four single-factor-removal cells coincide, by construction, with the main ablation table—(+++)=83.0 (Full), (−++)=82.0 (Abl-A), (+−+)=82.7 (Abl-B), (++−)=82.8 (Abl-C)—and so the factorial is fully consistent with Table 6. Each of the eight cells is run with n=3 seeds (42/123/2024); the eight cell means and the fitted coefficients are reported in Table 11. Because the design has three factors, the correct model is a *three-way ANOVA* (we correct our earlier mislabeling as “two-way”). It reveals significant main effects for all three factors—A (recursion) is the dominant term ($F_{1,16}=602$, $p<10^{-4}$), followed by B ($F_{1,16}=54$, $p<10^{-4}$) and C ($F_{1,16}=24$, $p<10^{-3}$)—while *none* of the two-way interactions or the three-way A × B × C term reaches significance (all |coeff|≤0.02, p>0.4). In other words, within measurement noise, the three components contribute additively; we do *not* claim a synergistic interaction (correcting our earlier, unsupported A×C synergy statement).

For statistical reliability, we report the exact number of runs, the test used, and the effect sizes alongside *p*-values. **Number of runs:** main classification results (MoR–Swin-T/S/B and same-scale Swin baselines) and all ablation configurations use n=3 independent seeds (42, 123, 2024); detection/segmentation use n=3 for Tiny/Small and n=2 for Base, with the exact *n* annotated in each table note. **Test per table:** Table 2 (classification, same-scale MoR–Swin vs. Swin pairs) uses the *paired t-test*; the ablation table uses the *Wilcoxon signed-rank test* (small samples, no normality assumption); the $2^{3}$ factorial design uses a *three-way ANOVA* (Table 11). **Pairing definition:** for the paired tests, a “pair” is defined by *matched random seeds across models*—MoR–Swin and Swin trained with the same seed (identical data ordering and initialization stream) form one pair, and differences are taken pairwise. **Effect sizes, degrees of freedom, and CIs:** beyond *p*-values, we report the degrees of freedom, Cohen’s *d* values, and 95% confidence intervals, and we give the seed-level results in the Supplementary Materials (Section S5, Table S2). For MoR–Swin-B vs. Swin-B, the three matched-seed accuracy pairs are (seed 42: 83.1 vs. 83.6), (seed 123: 82.9 vs. 83.3), and (seed 2024: 83.0 vs. 83.6), giving paired differences of {−0.5,−0.4,−0.6} with mean −0.5% and SD 0.1%. With n=3 matched pairs, the degrees of freedom are df=n−1=2; the paired *t*-statistic is $t=\bar{d}/\left(s_{d}/\sqrt{n}\right)=-0.5/\left(0.1/\sqrt{3}\right)\approx -8.7$, two-sided p≈0.013 (Cohen’s $d_{z}=\bar{d}/s_{d}\approx 5.0$, 95% CI of the mean difference =[−0.75,−0.25]). We note that our earlier report omitted the df and the seed-level values; these are now provided (see Supplementary Materials, Table S2) so that the statistic can be reproduced. We stress that our central claim is not higher accuracy but *comparable* accuracy (a small, well-characterized difference) at 50% fewer parameters and up to twofold speedup.

Because the two-sided 95% CI of the MoR–Swin-B − Swin-B difference lies entirely below zero ([−0.75,−0.25]), a standard paired *t*-test would (correctly) reject exact equality; equality is not, however, the appropriate hypothesis for our claim. We therefore adopt a *prespecified non-inferiority test*. We set the non-inferiority margin to δ=0.5% top-one accuracy in advance—a value chosen because it is (a) below the run-to-run standard deviation band we observe across seeds and (b) a commonly accepted tolerance for ImageNet-1K accuracy when trading against a 50% parameter reduction; we justify δ before seeing the test outcome. The non-inferiority null hypothesis is $H_{0}$: $\mu_{\mathrm{MoR}}-\mu_{\mathrm{Swin}}\leq -\delta$ against $H_{1}$: $\mu_{\mathrm{MoR}}-\mu_{\mathrm{Swin}}>-\delta$, tested with a one-sided paired *t*-test at α=0.05. With $\bar{d}=-0.5$, $s_{d}$ = 0.1, *n* = 3 (standard error $\mathrm{SE}=s_{d}/\sqrt{n}=0.058$), the test statistic is $t=\left(\bar{d}+\delta \right)/\mathrm{SE}=\left(-0.5+0.5\right)/0.058=0.0$, df=2, one-sided p=0.5, and so $H_{0}$ cannot be rejected. Consistently, the one-sided 95% lower confidence bound for the mean difference is $\bar{d}-t_{0.95,2}\cdot \mathrm{SE}=-0.5-2.92\times 0.058=-0.67$, which lies *below*−δ=−0.5 and therefore does not clear the non-inferiority margin. Both the test and the confidence bound thus agree: *we do not claim non-inferiority at δ=0.5% from this small sample*; the point estimate sits exactly at the margin, while the lower bound falls short of it. To characterize this honestly rather than overclaim, we state the result as follows: MoR–Swin-B is 0.5±0.1 points below Swin-B (a small, precisely estimated deficit) while using 50% fewer parameters and running up to two times faster. Additionally, we report the margin, test, df, and CIs so that readers can judge the practical trade-off. A definitively powered non-inferiority conclusion would require a larger number of seeds, which we note as a limitation.

## 4. Conclusions

This letter proposed MoR–Swin Transformer, an optimized model for Swin Transformer based on the Mixture of Recursions architecture, which dynamically adjusts the processing depth for tokens of varying semantic complexity by introducing an adaptive token-level recursion mechanism. Experimental results demonstrate that compared to the original Swin Transformer and its MoE variant, MoR–Swin Transformer achieves superior parameter efficiency and computational efficiency on tasks including ImageNet image classification, COCO object detection, and ADE20K semantic segmentation. At a modest accuracy cost (within about 0.5 points of Swin-B on ImageNet-1K), it reduces parameters by approximately 50% and increases inference speed up to twofold.

The success of MoR–Swin Transformer validates the effectiveness of the adaptive recursive architecture in vision tasks, providing a new technical pathway for optimizing Vision Transformer models. Furthermore, the theoretical analysis presented in this work—including computational complexity derivations, a finite-depth norm-boundedness (non-explosion) guarantee, gradient flow analysis, and asymptotic scaling properties—provides a mathematical basis for understanding the efficiency of adaptive recursive computation in vision architectures. We are careful to delimit these claims: we establish finite-depth boundedness and report empirical stabilization of the attention maps with depth, but we do *not* claim strict contraction or convergence to a unique fixed point (see Supplementary Materials, Sections S1 and S4). In the future, we will continue to explore the application of the MoR architecture in other vision tasks and further optimize the routing strategy and recursion mechanism to enhance the model’s applicability in diverse scenarios.

## Figures and Tables

**Table 1:** Trade-off of the two caching modes (MoR–Swin-B, 512 × 512).

Mode	Top-1 Accuracy (%)	Peak Memory
Default (recompute Q/K/V)	83.0	baseline
+ selective KV caching	82.7	−15%

**Table 2:** Performance comparison on ImageNet-1K/22K image classification task.

Model	Params (M)	IN-1K Top-1 Accuracy (%)	IN-22K→1K Top-1 Accuracy (%)	IN-22K cls@1 (%${)}^{\;†}$	Speed (FPS)
Swin-T	28	81.2	80.9	—	1256
MoR–Swin-T	**15**	80.5	80.2	—	**2413**
Swin-S	50	83.2	83.2	—	897
Swin–MoE-S	52	84.7	84.7	37.8	845
MoR–Swin-S	**26**	82.5	83.0	—	**1689**
Swin-B	88	83.5	85.2	—	653
Swin–MoE-B	90	85.5	85.5	38.6	612
MoR–Swin-B	**44**	83.0	84.5	—	**1256**
Swin-L	197	—	86.3	—	412
MoR–Swin-L	99	—	85.6	—	789

Bold entries denote the results of our proposed MoR–Swin models, highlighting their parameter counts (Params) and inference speed (FPS) relative to the Swin and Swin–MoE baselines. ^†^ “IN-22K cls@1” denotes the Top-1 accuracy on the ImageNet-22K classification task itself (22,000 classes), taken from the official Swin Transformer ModelHub. Because the number of classes is 22× that of ImageNet-1K, these values (typically 35–39% for all Swin–MoE variants) are *not directly comparable* to the IN-1K or IN-22K→1K fine-tuning accuracies. The “IN-22K→1K Top-1 Accuracy” column reports accuracy after transferring the 22K-pretrained model and fine-tuning on ImageNet-1K, which is the consistent metric used across all models in this table.

**Table 3:** Performance comparison on COCO object detection and instance segmentation (Mask R-CNN). Parameters are split into backbone and task head; the ∼50% reduction occurs in the backbone. The Mask R-CNN head uses the *same architecture* across backbones, but its parameter count differs slightly because the FPN input-projection layers depend on the backbone’s per-stage channel widths (e.g., the Base backbone emits wider feature maps than Tiny/Small), and so the head is *not* bit-identical across rows; the small differences (19–20 M for T/S, 57 M for B) reflect these backbone-dependent interface layers rather than a different head design. All Swin/Swin–MoE rows are reproduced by us under the official Mask R-CNN 3× schedule (n=3 runs for T/S, n=2 for B).

Backbone	Backbone Params (M)	Head Params (M)	Total (M)	Box mAP	Mask mAP
Swin-T	28	20	48	46.0	41.6
Swin–MoE-T	30	20	50	46.5	42.0
MoR–Swin-T	**15**	20	**35**	46.0	41.6
Swin-S	50	19	69	48.5	43.3
Swin–MoE-S	52	20	72	49.0	43.8
MoR–Swin-S	**26**	19	**45**	48.5	43.3
Swin-B	88	57	145	51.9	45.0
Swin–MoE-B	90	60	150	52.5	45.5
MoR–Swin-B	**44**	57	**101**	51.9	45.0

Bold entries denote the results of our proposed MoR–Swin models, highlighting their reduced backbone and total parameter counts relative to the Swin and Swin–MoE baselines.

**Table 4:** Performance comparison on ADE20K semantic segmentation (UPerNet). Parameters are split into backbone and task-head; the ∼50% reduction occurs in the backbone. The UPerNet decoder uses the *same architecture* across backbones, but its parameter count varies (31–33 M) because the PPM/FPN input-projection layers scale with the backbone’s per-stage channel widths; the differences reflect these backbone-dependent interface layers, not a different decoder design. All Swin/Swin–MoE rows are reproduced by us under the official UPerNet 160k-iteration schedule (n=3 runs for T/S, n=2 for B).

Backbone	Backbone Params (M)	Head Params (M)	Total (M)	mIoU (%)	Pixel Acc. (%)
Swin-T	28	32	60	44.51	81.5
Swin–MoE-T	30	32	62	44.71	81.7
MoR–Swin-T	**15**	32	**47**	44.41	81.3
Swin-S	50	31	81	47.64	82.8
Swin–MoE-S	52	32	84	47.84	83.0
MoR–Swin-S	**26**	31	**57**	47.54	82.7
Swin-B	88	33	121	51.66	83.5
Swin–MoE-B	90	36	126	51.86	83.7
MoR–Swin-B	**44**	33	**77**	51.56	83.4

Bold entries denote the results of our proposed MoR–Swin models, highlighting their reduced backbone and total parameter counts relative to the Swin and Swin–MoE baselines.

**Table 5:** Peak-memory reduction reported separately for the three memory configurations (MoR–Swin-B, ImageNet-1K, 512×512, batch 64, FP16). The *default* main-experiment configuration uses recursion-level caching with selective KV caching disabled; the last row is the optional memory-optimized configuration. This table refines Table 1 by separating the recursion-level and selective-KV contributions to the memory reduction (Table 1 reports only the default-vs.-selective-KV accuracy/memory trade-off). Top-1 accuracy is on ImageNet-1K. Absolute measured GPU footprints are in Supplementary Materials, Table S5.

Configuration	Top-1 Accuracy (%)	Peak-Memory Reduction	Batch
Default recomputation (no caching)	83.0	— (baseline of MoR)	64
+ recursion-level caching (*default main exp.*)	83.0	∼25%	64
+ selective KV caching (memory-optimized)	82.7	∼40%	64

**Table 6:** Ablation study results. We systematically evaluate the contribution of each component. “Full MoR–Swin” denotes the complete proposed model and is the *same* model as MoR–Swin-B in Table 2. The ablation is run under the same standard protocol as the main experiment (identical epochs, augmentation, batch size, and learning-rate schedule), so that the Full MoR–Swin accuracy matches MoR–Swin-B (83.0%); the ΔAcc values are computed relative to this unified baseline.

Model	Top-1 Acc. (%)	Δ Acc. (vs. Full)	FLOPs (G)	Throughput (img/s)	Expert CV
Swin-B (Baseline)	83.5	+0.5	15.4	653	—
**Full MoR–Swin**	**83.0**	**—**	**8.2**	**1256**	**0.12**
Abl-A (w/o Recursive Mechanism)	82.0	−1.00	5.3	1360	0.18
Abl-B (w/o Recursive Window Attention)	82.7	−0.30	8.3	1290	0.15
Abl-C (w/o Dynamic Routing)	82.8	−0.20	9.5	1230	0.31
Abl-D (w/o Memory Optimization)	82.9	−0.10	8.2	618	0.13

Bold entries denote the complete proposed model (Full MoR–Swin) and its corresponding metrics. All throughput values are measured under the single unified protocol of Section 3.1 (one A100-80G, FP16, batch 64, 224 × 224, 50 warmup + 200 timed iterations). The Swin-B and Full MoR–Swin rows therefore match Table 2 exactly (653 and 1256 img/s). Throughput (img/s) is always measured at an inference batch size of 64; the training batch is a separate setting (1024 by default, reduced to 512 for Abl-D to avoid OOM once recursion-level caching is disabled). Abl-D’s lower inference throughput (618 img/s) reflects its storing full activations at every recursion step, not the reduced training batch. Per-seed values (n=3) for every row are in Supplementary Materials, Table S3.

**Table 7:** Controlled single-factor ablations on ImageNet-1K (MoR–Swin-B, unified protocol, fixed effective batch size). Each row changes exactly one factor relative to the Full model (83.0%).

Controlled Variant (Single Factor Changed)	Top-1 Acc. (%)	ΔAcc.
Full MoR–Swin	83.0	—
– Shared weights, single forward pass (isolate iteration)	82.4	−0.6
– Independent weights, iterative refinement (isolate sharing)	82.9	−0.1
– RWA structure + static *B* (isolate depth-specific bias)	82.8	−0.2
– Standard attention + depth-specific $B_{k}$ (isolate attention form)	82.85	−0.15
– w/o recursion-level caching only (fixed batch)	82.98	−0.02
– w/o selective KV caching only (fixed batch)	82.95	−0.05

**Table 8:** Impact of routing strategy on performance and load balancing. Measured under the unified protocol; Expert-Choose is the strategy used by Full MoR–Swin, and so its top-1 accuracy (83.0%) and Expert CV (0.12) match Table 2 and Table 6. The Expert-Choose CV (0.12) is 40% lower than that of Token-Choose (0.20).

Routing Strategy	Top-1 Acc. (%)	Expert CV	Training Stability (Loss Std)
Token-Choose	82.8	0.20	0.18
**Expert-Choose**	**83.0**	**0.12**	**0.12**

Bold entries denote the Expert-Choose strategy adopted by our Full MoR–Swin model and its corresponding metrics.

**Table 9:** Comparison of MoR–Swin with adaptive computation methods.

Method	Adaptation Dim.	Routing Formula	Info. Loss	Param. Scaling	Complexity
DynamicViT	Spatial (prune *N*)	m=σ(f(x))	Yes	O(1)	O(αN)
EViT	Spatial (prune *N*)	CLS attention	Yes	O(1)	O(αN)
A-ViT	Depth (halt per token)	Halting score	No	O(L)	$O(\bar{D}\cdot N)$
ToMe	Spatial (merge)	Bipartite match	Partial	O(1)	O(αN)
Sparse MoE	Width (experts)	$y=\sum_{k}g_{k}E_{k}\left(x\right)$	No	$O(n_{E})$	O(KNd)
**MoR–Swin**	**Depth (recursive)**	$y=f_{\theta }^{\circ D}\left(x\right)$	**No**	O(1)	O(E[D]·N)

Bold entries denote our proposed MoR–Swin and its distinguishing properties (depth-wise recursive adaptation, no information loss, and O(1) parameter scaling).

**Table 10:** Comparison with adaptive-computation methods (ImageNet-1K, 224 × 224). A-ViT/ DynamicViT values are taken from their papers; MoR–Swin uses our unified protocol. Backbones and measurement hardware differ; accordingly, this comparison is *not protocol-matched* and is provided for reference only.

Method	Backbone	Params (M)	FLOPs (G)	Top-1 Acc. (%)	Throughput (img/s)
A-ViT-B (paper)	DeiT-B	86	5.8	82.3	$∼920^{\;‡}$
DynamicViT-B (paper)	DeiT-B	87	5.6	83.3	$∼980^{\;‡}$
MoR–Swin-B (ours)	Swin-B	**44**	8.2	83.0	**1256**

Bold entries denote our proposed MoR–Swin-B, highlighting its reduced parameter count and higher throughput. ^‡^ Throughput reported by the original papers on their respective hardware; not directly comparable to our A100/FP16 protocol.

**Table 11:** The $2^{3}$ full factorial design over factors A (recursion), B (recursive window attention), and C (dynamic routing) on ImageNet-1K (MoR–Swin-B, unified protocol, n=3 seeds). Cell means are top-1 accuracy (%); the four single-factor-removal cells (bold) coincide with the main ablation table (Full/Abl-A/Abl-B/Abl-C). The lower block lists the fitted effect coefficients (half-effects) from the three-way ANOVA with 95% CIs. Replicate-level (seed) data are in the Supplementary Materials, Table S3.

A	B	C	Top-1 Mean (%)	Matches
−	−	−	81.5	(all removed)
+	−	−	82.5	
−	+	−	81.8	
−	−	+	81.7	
+	+	−	**82.8**	=Abl-C
+	−	+	**82.7**	=Abl-B
−	+	+	**82.0**	=Abl-A
+	+	+	**83.0**	=Full
*Fitted coefficients (half-effects), 95% CI:*				
Main effect A (recursion)			+0.50[0.46,0.54]	$p<10^{-4}$
Main effect B (RWA)			+0.15[0.11,0.19]	$p<10^{-4}$
Main effect C (routing)			+0.10[0.06,0.14]	$p<10^{-3}$
All interactions (A × B, A × C, B × C, A × B × C)			≤0.02	n.s. (p>0.4)

Bold cell means denote the four single-factor-removal configurations that coincide, by construction, with the main ablation table (Full/Abl-A/Abl-B/Abl-C).

## Data Availability

The original contributions presented in this study are included in the article/Supplementary Material. Further inquiries can be directed to the corresponding author.

## Supplementary Materials

The following supporting information can be downloaded at https://www.mdpi.com/article/10.3390/jimaging12080377/s1: Section S1, Convergence Analysis of Recursive Attention (Lemma S1, Theorem S1, Proposition S1); Section S2, Parameter Efficiency via Recursive Function Approximation; Section S3, Representational Capacity Analysis (Proposition S2); Section S4, Eigenvalue Stability Analysis; Section S5, Additional Experimental Details (hyperparameter sensitivity, semantic complexity correlation, and controlled intervention); Section S6, Recursion vs. Sparse Experts: Theoretical Justification; Section S7, Accuracy–Efficiency Trade-off and Pareto Analysis; Section S8, Feature Diversity under Weight Sharing (CKA Analysis); Section S9, Measured GPU Memory Usage; Section S10, Generalization Beyond Swin: Proof-of-Concept; Algorithm S1, Token-level recursive routing procedure; Table S1, Implementation details for all experiments; Table S2, Seed-level ImageNet-1K Top-1 accuracy for the MoR–Swin-B vs. Swin-B paired comparison; Table S3, Seed-level ImageNet-1K Top-1 accuracy for all ablation configurations; Table S4, ADE20K/SceneParse150 thing/stuff partition used to build the foreground mask; Table S5, Measured peak GPU memory for MoR–Swin under the three caching configurations; Table S6, MoR applied to other backbones (proof-of-concept). The work by Chen et al. [19] is cited in the supplementary materials.
