# Supplementary material for: MoR–Swin: Efficient Vision Transformer Using Mixture of Recursions
Source: J Imaging. 2026 Aug 12;12(8):377. doi: 10.3390/jimaging12080377 (PMC13514322; doi:10.3390/jimaging12080377)
Supplement: Supplementary file 1 [file jimaging-12-00377-s001.zip › jimaging-4407936-supplementary.pdf]

Supplementary Materials: MoR-Swin: Efficient Vision Transformer via Mixture of Recursions—Supplementary Material

Yongbao Ai [1,*], TianXiang Gao [1], Zhipeng Lin [1], Longqi Yang [1] and Qingyu Chang [1]

This Supplementary Material provides the deeper theoretical proofs referenced in the main text (Comments 4, 10, 14, 17 from Reviewer 1). It also includes hyperparameter sensitivity analysis and semantic complexity correlation experiments.

S1. Convergence Analysis of Recursive Attention

Consider the single-step recursive operator:

$$T(z) = z + \mathrm{RWA}(\mathrm{LN}(z)). \tag{S1}$$

We establish that repeated application of T does not cause the feature representations to explode.

Lemma S1 (Bounded Increment). For any input $z \in \mathbb{R}^{N\times d}$, the increment at each recursion step satisfies:

$$\|\mathrm{RWA}(\mathrm{LN}(z))\|_F \leq \|A\|_2 \cdot \|W_V\|_2 \cdot \sqrt{Nd}, \tag{S2}$$

where A is the attention matrix, W_V is the value projection matrix, and $\|\cdot\|_F$ denotes the Frobenius norm.

Proof. Layer normalization constrains the norm of each token: $\|\mathrm{LN}(z_i)\|_2 = \sqrt{d}$ for all i. Thus $\|\mathrm{LN}(z)\|_F = \sqrt{Nd}$.

The attention operation computes $\mathrm{Attn}(\cdot) = A \cdot \mathrm{LN}(z) \cdot W_V$, where $A \in \mathbb{R}^{N\times N}$ is the row-stochastic attention matrix (each row sums to 1). We note that row stochasticity implies $\|A\|_\infty = 1$ and spectral radius $\rho(A) = 1$, but *not* $\|A\|_2 \leq 1$ in general; we therefore retain $\|A\|_2$ explicitly rather than bounding it by 1. By submultiplicativity:

$$\|\mathrm{RWA}(\mathrm{LN}(z))\|_F = \|A \cdot \mathrm{LN}(z) \cdot W_V\|_F \leq \|A\|_2 \cdot \|\mathrm{LN}(z)\|_F \cdot \|W_V\|_2 = \|A\|_2 \cdot \|W_V\|_2 \cdot \sqrt{Nd}. \tag{S3}$$

Empirically, we measured $\|A\|_2 \approx 1.0$–1.3 across layers, so the per-step increment remains bounded by a moderate constant. □

Theorem S1 (Non-Explosion Guarantee). After N_a recursion steps starting from z_0, the feature norm satisfies:

$$\|z_{N_a}\|_F \leq \|z_0\|_F + N_a \cdot c_{\max} \cdot \sqrt{Nd}, \qquad c_{\max} = \max_k \|A_k\|_2 \|W_V\|_2. \tag{S4}$$

Thus the features grow at most linearly with recursion depth, preventing exponential blow-up.

Proof. By the triangle inequality and Lemma S1: $\|z_{k+1}\|_F = \|z_k + \mathrm{RWA}(\mathrm{LN}(z_k))\|_F \leq \|z_k\|_F + \|A_k\|_2\|W_V\|_2\sqrt{Nd} \leq \|z_k\|_F + c_{\max}\sqrt{Nd}$. Applying this recursively for $k = 0, \ldots, N_a - 1$ yields the result. The bound is linear (not strictly $\leq \sqrt{Nd}\|W_V\|_2$ per step, since $\|A\|_2$ is not guaranteed to be 1); with the measured $c_{\max} \approx 1.1$–1.4 and small $N_a \leq 5$, the accumulated growth stays bounded and controlled. □

Proposition S1 (Sufficient condition for contraction — caveat). One might hope that introducing a residual scaling α in $T_\alpha(z) = z + \alpha\,\mathrm{RWA}(\mathrm{LN}(z))$ makes T_α a contraction. However, we caution that the condition $\alpha\|W_V\|_2 < 1$ is *not* sufficient. Because T_α contains

the identity term, its Jacobian is $I + \alpha J_F$ (with J_F the Jacobian of $F = \text{RWA} \circ \text{LN}$), and contraction requires $\|I + \alpha J_F\|_2 < 1$—i.e., the spectrum of αJ_F must lie strictly inside the unit disk centered at -1. Merely bounding the Lipschitz constant of F by $\alpha\|W_V\|_2 < 1$ controls $\|\alpha J_F\|$ but does not place its eigenvalues in that disk, so a strict contraction (and hence a unique Banach fixed point) is *not* established. We therefore do not claim convergence to a unique fixed point; we claim only boundedness (Theorem S1) and empirical stabilization (below).

Remark (Applicability to the deployed model). We emphasize that the residual scaling factor α is *not* part of the deployed architecture: our trained and evaluated MoR-Swin uses standard residual connections ($\alpha = 1$). As noted in Proposition S1, we do not claim a strict contraction even for the scaled variant. What holds unconditionally for the deployed model ($\alpha = 1$) is Theorem S1 (non-explosion): the feature norm grows at most linearly with depth and is therefore bounded, which suffices for numerical stability at the small recursion depths used in practice ($N_a \leq 5$). Empirically, we measured the spectral norm $\|W_V\|_2$ of the value-projection matrices of the trained model: across the four stages it lies in the range 0.8–1.4 (mean ≈ 1.1). Thus for the deployed model $\alpha\|W_V\|_2 \approx 1.1$, slightly above 1, placing it at the boundary of—rather than strictly inside—the contraction regime. This is consistent with our observation that the recursion produces a progressively refined, bounded feature sequence rather than converging to a strict fixed point. The monotonically decreasing JSD between consecutive attention maps (Section 3.7 of the main text; from 0.142 to 0.058) provides empirical evidence that, at the actual operating point ($\alpha = 1$, $\|W_V\|_2 \approx 1.1$), the attention distributions nonetheless stabilize with depth.

S2. Parameter Efficiency via Recursive Function Approximation

Parameter sharing across recursion steps constrains N_a independent layer mappings $\{f_{\theta_1}, \ldots, f_{\theta_{N_a}}\}$ into N_a compositions of a single mapping f_θ:

$$f_\theta^{\circ N_a}(z) = \underbrace{f_\theta \circ f_\theta \circ \cdots \circ f_\theta}_{N_a \text{ times}}(z). \tag{S5}$$

S2.1. ODE Interpretation

The residual iteration $z_{k+1} = z_k + f_\theta(z_k)$ is the forward-Euler discretization (step size $h = 1$) of the ordinary differential equation:

$$\frac{dz}{dt} = f_\theta(z), \quad z(0) = z_0. \tag{S6}$$

Sharing θ means we integrate the *same autonomous vector field* for N_a steps. This is analogous to Neural ODEs, but with adaptive, token-dependent integration lengths D_i.

S2.2. Approximation Capacity

Under the assumption that f_θ is L-Lipschitz continuous with $L < \infty$, the N_a-step shared iteration can approximate any smooth transformation $\Phi : \mathbb{R}^d \to \mathbb{R}^d$ that is reachable as the time-N_a flow of a Lipschitz vector field, with approximation error bounded by the Euler discretization error $O(h^2 L^2 N_a / 2)$.

S2.3. Parameter Count Comparison

- **Independent layers**: N_a layers each with $|\theta|$ parameters $\Rightarrow$ total $N_a|\theta|$.
- **Shared recursion**: 1 shared block $\Rightarrow$ total $|\theta| + N_a|B_k|$, where $|B_k| \ll |\theta|$ (only position biases are depth-specific).

The parameter reduction ratio is approximately $1/N_a$ (or equivalently $1/L$ per stage, as derived in Section 2.6 of the main text), achieving ∼50% reduction overall for MoR-Swin-B.

S3. Representational Capacity Analysis

Proposition S2. The shared-parameter N_a-fold recursion $f_\theta^{\circ N_a}$ strictly increases the hypothesis class relative to a single application f_θ.

Argument. The composition $f_\theta^{\circ N_a}$ can represent functions that f_θ alone cannot. For example, if f_θ implements a contraction by factor $c < 1$, then $f_\theta^{\circ N_a}$ contracts by c^{N_a}—a qualitatively different transformation unreachable by f_θ applied once. More generally, the iterated map $z \mapsto z + f_\theta(z + f_\theta(\cdots))$ creates nested nonlinearities of degree growing with N_a.

Limitation. The shared-parameter recursion does *not* achieve the full expressiveness of an independently-parameterized N_a-layer network, because it is constrained to the orbit of a single vector field. It forms a strict subset of the mappings expressible by N_a independent layers. This is the fundamental trade-off: parameter efficiency (shared θ) in exchange for a restricted (but still enriched) hypothesis class.

Empirical evidence: In the ablation study (Table 6 of the main text), removing the recursion mechanism (Abl-A) causes a 1.0% accuracy drop, confirming that recursion provides genuine expressiveness gains beyond what can be achieved by the base single-pass architecture.

S4. Eigenvalue Stability Analysis

To analyze whether the recursive feature transformation neither explodes nor vanishes, we study the spectrum of the linearized recursion operator.

S4.1. Linearized Recursion

At a fixed point or along a trajectory, linearize the recursion:

$$z_{k+1} = z_k + f_\theta(z_k) \approx (I + J_k)z_k, \tag{S7}$$

where $J_k = \frac{\partial f_\theta}{\partial z}\Big|_{z=z_k}$ is the Jacobian of the recursion function at step k.

S4.2. Stability Conditions

For the recursion to be stable (non-exploding, non-vanishing), the spectral radius of $(I + J_k)$ must satisfy:

$$\rho(I + J_k) \approx 1. \tag{S8}$$

Non-amplification ($|1 + \lambda_i| \leq 1$) requires each eigenvalue $\lambda_i(J_k)$ (in general complex) to lie in the closed unit disk centered at -1:

$$(\mathrm{Re}(\lambda_i) + 1)^2 + (\mathrm{Im}(\lambda_i))^2 \leq 1 \quad \forall\, i. \tag{S9}$$

We stress that constraining only the real part (e.g., $-2 < \mathrm{Re}(\lambda_i) < 0$) is *not* sufficient for complex eigenvalues, since a large imaginary part can push $|1 + \lambda_i|$ above 1; the correct condition is the disk condition above.

S4.3. Why MoR-Swin Satisfies These Conditions

1. **Layer Normalization**: LN projects inputs onto a hypersphere of radius $\sqrt{d}$, bounding the input magnitude to the attention operator and preventing unbounded growth of the Jacobian.

2. **Row-Stochastic Attention**: The attention matrix A is row-stochastic, so $\|A\|_\infty = 1$ and its spectral radius is $\rho(A) = 1$ (with the all-ones right eigenvector); note, however, that A may still have complex eigenvalues of modulus up to 1 and $\|A\|_2$ is not bounded by 1 in general. This bounds—but does not by itself pin down—the spectral radius of the attention component of J_k; we therefore treat it as one contributing factor rather than a guarantee.
3. **Residual Scaling**: With scaling factor α, the Jacobian becomes $I + \alpha J_k$. We caution that a *small* α does *not* by itself guarantee non-amplification: bounding $\|\alpha J_k\|_2$ (or constraining only $\mathrm{Re}(\lambda_i)$) controls magnitude but does not place the eigenvalues of αJ_k inside the unit disk centered at -1 required by the disk condition above; eigenvalues with a large imaginary part, or with positive real part, can still yield $|1 + \alpha\lambda_i| > 1$. Hence residual scaling should be read as an empirical stabilizer rather than a sufficient condition for stability, consistent with Proposition S1.

Scope of the claims. To avoid overstatement, we distinguish four increasingly strong properties: (i) *finite-depth norm boundedness* (Theorem S1), which holds *unconditionally* for the deployed model ($\alpha = 1$, $N_a \leq 5$); (ii) *non-amplification* ($|1 + \lambda_i| \leq 1$), which requires the disk condition and is only observed *empirically* (below); (iii) *contraction*, which additionally requires $\|I + \alpha J_F\|_2 < 1$ and is *not* established (Proposition S1); and (iv) *convergence to a unique fixed point*, which we do *not* claim. Our theoretical guarantee is limited to (i); (ii) is an empirical finding; (iii)–(iv) are explicitly disclaimed.

S4.4. Empirical Verification

We compute the eigenvalue spectrum of the Jacobian J at representative layers during inference on ImageNet validation images. The eigenvalues consistently cluster near the origin with $|\mathrm{Re}(\lambda)| < 0.3$ and $|\mathrm{Im}(\lambda)| < 0.5$, confirming that $(I + J)$ has spectral radius close to 1 and the recursion remains in the stable regime for all observed inputs up to $N_a = 5$.

S5. Additional Experimental Details

S5.1. Implementation Details

Table S1 lists the complete set of training and routing hyperparameters used in all experiments, ensuring full reproducibility.

Table S1. Implementation details for all experiments.

Item	Setting
Optimizer	AdamW (β_1=0.9, β_2=0.999, wd 0.05)
Learning rate	5×10^{-4} (cosine decay), 20-epoch warmup
Epochs	300 (cls), 36 (det 3×), 160k iter (seg)
Batch size	1024 (cls), 16 (det/seg)
Resolution	224×224 (cls), 512×512 (seg)
Augmentation	RandAug, Mixup (α=0.8), CutMix (α=1.0)
Seeds	3 runs (42, 123, 2024)
Selection fraction r	0.8 (per-stage token proportion)
Max recursion N_a	3 (cls), 5 (seg)
Balance λ_b	0.01 (Tiny/Small), 0.01/0.005 (Base), 0.005 (Large)
Target t_i	t_1=0.8, t_2=0.5, t_3=0.3
Threshold θ	0.6
Gumbel τ	5.0 $\rightarrow$ 0.5 (anneal 100 epochs)
Detection	Mask R-CNN 1×/3×, Swin official
Segmentation	UPerNet 160k iter, Swin official

S5.2. Routing Procedure Pseudocode

Algorithm S1 specifies the complete token-level recursive routing procedure, making explicit how scoring, top-fraction/Expert-Choose selection, STE/Gumbel-Softmax, threshold-based stopping, and recursion are combined during training and inference. The token selection weight w (Eq. 1) and the per-token depth distribution $p_{i,k}$ (Eq. 3) are computed once, so the threshold stopping rule (Eq. 4) is dimensionally consistent ($\sum_k p_{i,k} = 1$).

Algorithm S1: Token-level recursive routing (per stage).

Input: tokens $x = \{x_i\}_{i=1}^{N}$, $x_i \in \mathbb{R}^C$; max depth N_a; selection fraction r, per-step capacity $C_{\text{cap}} = \lfloor rN \rfloor$; depth-probability threshold θ; mode $\in$ {train, infer}.

Notation: s_i = per-token selection score; w = token-normalized selection weights (over i, Eq. 1); $g_{i,k}$ = depth scores and $p_{i,k}$ = depth-normalized probabilities (over $k = 1..N_a$, Eq. 3), *computed once* per token; D_i = per-token target depth ($D_i = 0$ for bypassed tokens, else $D_i \in \{1, .., N_a\}$ from Eq. 4); $m_i^{(t)} \in \{0, 1\}$ = gate indicating token i is active at step t.

// Stage 1: score once, select the recursion set, set per-token target depth

1: $s_i \leftarrow \text{Linear}_{C\to 1}(x_i)$; $w \leftarrow \text{Softmax}_i(\{s_i\}_{i=1}^{N})$ (token-normalized selection, Eq. 1)

2: $\mathcal{R} \leftarrow \text{TopK}(w, C_{\text{cap}})$ (the $\lfloor rN \rfloor$ selected tokens that may recurse; Expert-Choose)

3: $g_{i,k} \leftarrow \text{Linear}_{C\to N_a}(x_i)$; $p_{i,k} \leftarrow \text{Softmax}_k(\{g_{i,k}\}_{k=1}^{N_a})$ (depth distribution, Eq. 3)

4: **for** each token i: **if** $i \in \mathcal{R}$ **then** $D_i \leftarrow \min\{t \in \{1, .., N_a\} : \sum_{k\le t} p_{i,k} \ge \theta\}$ **else** $D_i \leftarrow 0$ (non-selected tokens bypass entirely)

// Stage 2: recursive refinement up to each token's own depth

5: $z^{(0)} \leftarrow x$

6: **for** $t = 0$ **to** $N_a - 1$ **do**

7: $m_i^{(t)} \leftarrow \mathbb{1}[t < D_i]$ (active while below target depth; since only the $\lfloor rN \rfloor$ tokens in $\mathcal{R}$ have $D_i \ge 1$, the active count $\sum_i m_i^{(t)} \le C_{\text{cap}}$ at every step)

8: **if** mode = train: apply STE $m_i^{(t)} \leftarrow \tilde{m}_i^{(t)} + (m_i^{(t)} - \tilde{m}_i^{(t)})_{\text{stop-grad}}$, $\tilde{m}^{(t)}$ from Gumbel-Softmax(s, τ) (backward soft; forward hard)

9: active i ($m_i^{(t)}$=1): $z_i^{(t+1)} \leftarrow z_i^{(t)} + \text{RWA}_t(\text{LN}(z_i^{(t)}))$; else $z_i^{(t+1)} \leftarrow z_i^{(t)}$; cache KV of active tokens only

10: **Output:** refined tokens $z_i^{(\max(D_i,0))}$ (bypassed tokens with D_i=0 return $z_i^{(0)}$=x_i) and per-token depths D_i

Figure S1. Pseudocode of the token-level recursive routing used in training and inference (Algorithm S1).

S5.3. Seed-Level Results and Statistical Details

Table S2 reports the per-seed Top-1 accuracy for the paired comparison in the main text (Section on Statistical Analysis). Pairing is defined by matched random seeds across models. With $n = 3$ matched pairs, the degrees of freedom are $df = 2$; the paired t-test gives $t \approx -8.7$, two-sided $p \approx 0.013$, Cohen's $d_z \approx 5.0$, 95% CI of the mean difference $= [-0.75, -0.25]$. Full seed-level results for all configurations and the code/config files are provided in the released repository for independent verification.

Table S2. Seed-level ImageNet-1K Top-1 accuracy (%) for the MoR-Swin-B vs. Swin-B paired comparison. Table numbered S2 in the Supplementary Material.

Model	Seed 42	Seed 123	Seed 2024	Mean ± SD
Swin-B	83.6	83.3	83.6	83.5 ± 0.17
MoR-Swin-B	83.1	82.9	83.0	83.0 ± 0.10
Paired diff.	−0.5	−0.4	−0.6	−0.5 ± 0.1 (df=2, t≈−8.7, p≈0.013)

Table S3. Seed-level ImageNet-1K Top-1 accuracy (%) for all ablation configurations of main-text Table 6, measured under the unified protocol ($n = 3$ seeds). Mean values match the main-text table; standard deviations quantify the run-to-run variability relevant to the 0.02–0.05-point differences in the controlled ablations.

Configuration	Seed 42	Seed 123	Seed 2024	Mean ± SD
Full MoR-Swin	83.1	82.9	83.0	83.0 ± 0.10
Abl-A (w/o recursion)	82.1	81.9	82.0	82.0 ± 0.10
Abl-B (w/o RWA)	82.8	82.6	82.7	82.7 ± 0.10
Abl-C (w/o routing)	82.9	82.7	82.8	82.8 ± 0.10
Abl-D (w/o memory opt.)	83.0	82.8	82.9	82.9 ± 0.10
Controlled single-factor ablations (Table 7):				
Isolate iteration	82.5	82.3	82.4	82.4 ± 0.10
Isolate sharing	83.0	82.8	82.9	82.9 ± 0.10
Static B (isolate bias)	82.9	82.7	82.8	82.8 ± 0.10
Std. attn. + depth B_k	82.95	82.75	82.85	82.85 ± 0.10
w/o recursion-level caching	83.08	82.88	82.98	82.98 ± 0.10
w/o selective KV caching	83.05	82.85	82.95	82.95 ± 0.10

The ~0.10 standard deviation across seeds is comparable to (or larger than) the 0.02–0.05-point differences among the caching variants, confirming that those differences are within run-to-run noise and should not be over-interpreted; the iteration effect (−0.6) and the recursion effect (−1.0) are, by contrast, well outside the noise band.

S5.4. Hyperparameter Sensitivity

- λ_b (load balance weight): set to 0.01 for Tiny/Small (all stages), 0.01 for stages 1–3 and 0.005 for stage 4 in Base, and 0.005 for all stages in Large. Sensitivity tested in $\{0.001, 0.01, 0.1, 1.0\}$: performance is robust for $\lambda_b \in [0.005, 0.1]$; $\lambda_b > 1.0$ slightly degrades task accuracy; $\lambda_b < 0.001$ leads to load imbalance (CV > 0.3). Larger models benefit from smaller λ_b because their greater capacity is less sensitive to load-balancing regularization.
- λ_c (recursive consistency): fixed at 0.05 across all scales (consistent with Section 3 of the main text); helps stabilize training in the first 50 epochs, can be annealed to 0 after convergence. (The previously listed ℓ_1 routing-sparsity weight λ_r has been removed, since $\|w\|_1 \equiv 1$ for a Softmax output makes that term ineffective; see the main-text objective.)
- Temperature τ for Gumbel-Softmax: initial 5.0, linearly annealed to 0.5 over 100 epochs, then fixed.

S5.5. Semantic Complexity Correlation

We compute the Spearman rank correlation between the attention entropy $\mathcal{C}_i$ and the actual recursion depth D_i assigned by the trained router. The attention entropy $\mathcal{C}_i$ is best described as a *computable proxy correlated with* semantic complexity—it captures contextual uncertainty, which is strongly associated with, but not identical to, semantic complexity.

Cross-task correlation. We report the correlation across three tasks: ImageNet classification ($\rho = 0.72$, $p < 10^{-6}$, $n = 10{,}000$ images), ADE20K segmentation ($\rho = 0.69$, $p < 10^{-6}$), and COCO detection ($\rho = 0.66$, $p < 10^{-6}$). The consistent range of 0.66–0.72 across tasks indicates that the association between high-entropy tokens and deeper recursion is robust rather than ImageNet-specific. Alternative complexity measures on ImageNet yield: feature variance ($\rho = 0.61$), inverse token confidence ($\rho = 0.68$).

Confound analysis. Attention entropy may correlate with factors other than semantic complexity per se; we examine two: (1) *Token position*—central tokens tend to have higher

entropy. Controlling for the normalized distance of a token to the image center via partial correlation yields $\rho_{\text{partial}} = 0.64$ (vs. 0.72 unconditioned), so position is not the dominant driver. (2) *Object scale*—small-object tokens tend to have higher entropy. Stratifying by object scale (small/medium/large, using COCO area thresholds), the correlation remains in the 0.60–0.70 range within each stratum, indicating the association is not driven by scale alone. In light of these confounds, we use cautious wording throughout ("$\mathcal{C}_i$ is a proxy correlated with semantic complexity", not "$\mathcal{C}_i$ measures semantic complexity"). The recursion-depth vs. segmentation-foreground IoU of 0.68 reported below provides spatial evidence independent of entropy, further supporting that deep recursion concentrates on semantically rich regions.

To further verify that deep recursion corresponds to visually complex regions, we compute the overlap between the recursion-depth heatmap and semantic segmentation ground truth on 1,000 ADE20K validation images. Specifically, we binarize the depth map at the median depth (tokens above median = "complex") and compare against a foreground mask derived from the segmentation annotation. **Foreground-mask construction:** following the ADE20K/SceneParse150 convention, we partition the 150 categories into "stuff" classes (amorphous background regions such as sky, wall, floor, road, grass) and "things" classes (countable object instances such as person, car, chair); the foreground mask is the union of all "things"-class pixels, downsampled to the token grid via majority voting within each patch. The resulting mean IoU between the high-recursion region and this foreground mask is 0.68, indicating that deep recursion concentrates on object-rich regions while shallow recursion falls on background. This provides spatial evidence complementing the scalar Spearman correlation above. **The complete thing/stuff category partition is given in Table S4.**

Table S4. ADE20K/SceneParse150 thing/stuff partition used to build the foreground mask. "Stuff" (background) = amorphous regions without well-defined instances; "things" (foreground) = countable object instances. The mask is the union of all "things"-class pixels. Representative classes are listed; the full 150-class assignment is released with the code.

Group	#classes	Representative categories
Stuff (background)	35	sky, wall, floor, ceiling, road, grass, earth, sidewalk, sea, river, mountain, field, sand, water, path, runway, snow, hill, dirt-track, land
Things (foreground)	115	person, car, chair, table, sofa, bed, lamp, cabinet, door, window, plant, curtain, painting, book, cushion, box, flower, pot, bottle, vase, tv, computer, bicycle, boat, bus, truck, traffic-light, sign, clock

Controlled intervention (does depth *benefit* complex tokens?). Correlation alone cannot show that high-complexity tokens *benefit* from additional recursion. We therefore intervene at inference on the trained model: (a) *force-shallow*—cap the recursion depth of the top-25% highest-entropy tokens to $D = 1$; (b) *force-deep*—raise the depth of the bottom-25% lowest-entropy tokens to $D = N_a$. Relative to the unmodified model (83.0 $\pm$ 0.10%, mean $\pm$ SD over seeds 42/123/2024), force-shallow drops Top-1 to 82.1 $\pm$ 0.12% ($\Delta = -0.9 \pm 0.05\%$), while force-deep changes it negligibly to 83.0 $\pm$ 0.11% ($\Delta = +0.0 \pm 0.04\%$) at higher cost. The per-seed force-shallow drops are $\{-0.85, -0.94, -0.91\}$ and the force-deep changes are $\{+0.02, -0.03, +0.01\}$, so the asymmetry is consistent across all three seeds (the force-shallow effect exceeds its SD by $> 15\times$, whereas force-deep is within noise). This asymmetry—removing depth from high-entropy tokens hurts, adding depth to low-entropy tokens does not help—provides evidence, stronger than correlation, that the

learned routing allocates useful computation to high-complexity tokens. We nonetheless state the overall conclusion as an association supported by this intervention, not as a proof of a causal "semantic complexity" mechanism.

S5.6. Selection Fraction r and N_a Sensitivity

- Selection fraction r (proportion of tokens entering recursion, $\lfloor rN \rfloor$ tokens per stage): tested in $\{0.5, 0.7, 0.8, 0.9\}$. Optimal at $r = 0.8$ for classification (balances efficiency and accuracy, and satisfies the constraint $r \geq t_1 = 0.8$ so the first-recursion load target is attainable). Below 0.7, too few tokens refine and accuracy drops (by 0.8% at $r = 0.5$); at $r = 0.9$ efficiency gains diminish with no accuracy benefit.
- Maximum recursion depth N_a: classification optimal at 3 (accuracy saturates beyond 3, FLOPs continue rising); segmentation optimal at 5 (complex scene parsing benefits from deeper refinement).
- Routing threshold θ: tested in $\{0.3, 0.5, 0.7, 0.9\}$. Performance is robust for $\theta \in [0.5, 0.7]$; below 0.3, most tokens exit too early (accuracy -0.6%); above 0.9, nearly all tokens recurse to N_a (losing the efficiency benefit).

S6. Recursion vs. Sparse Experts: Theoretical Justification

Swin Transformer's hierarchical window attention already reduces per-layer complexity to $O(NM^2d)$, where M is the window size. The remaining efficiency bottleneck is *depth stacking*: each additional layer adds $\sim 12d^2$ parameters and requires storing activations for all N tokens. Sparse MoE addresses this by expanding capacity along the width dimension (multiple expert sub-networks), but introduces: (i) parameter growth proportional to the number of experts, (ii) all-to-all communication in distributed settings, and (iii) load imbalance requiring auxiliary losses.

Recursion addresses the same bottleneck differently. The update $z_{k+1} = z_k + f_\theta(z_k)$ reuses a *single* parameter set θ for N_a steps—a forward-Euler discretization of the ODE $\dot{z} = f_\theta(z)$. This yields:

- **Near-constant parameters**: one shared block replaces L independent blocks ($\sim 1/L$ parameter ratio).
- **No inter-device communication**: all recursion happens locally within each window, unlike MoE's expert-parallel routing.
- **Natural match to spatial heterogeneity**: per-token variable depth D_i allocates compute where needed, while MoE allocates compute uniformly across selected experts regardless of spatial structure.

For hierarchical window attention specifically, the local (non-overlapping) window structure means each recursion step refines a bounded spatial context—ideal for the ODE-style iterative refinement where each step builds on the previous one within the same receptive field.

S7. Accuracy–Efficiency Trade-off and Pareto Analysis

To quantify the accuracy-vs-efficiency trade-off, we plot Accuracy vs. FLOPs and Accuracy vs. Latency curves for MoR-Swin, Swin, and Swin-MoE across all four scales (Tiny, Small, Base, Large). The key observations are:

1. **Pareto dominance**: All accuracies in this analysis are standard ImageNet-1K Top-1 (not IN-22K→1K), consistent with the main-text ImageNet-1K/22K classification table, and all FLOPs are at 224×224 (Swin-T/S/B $\approx$ 4.5/8.7/15.4 G; MoR-Swin-T/S/B $\approx$ 2.4/4.6/8.2 G, i.e. about $0.53\times$ the same-scale Swin, following $\mathbb{E}[D]/N_a$). At an equivalent FLOPs budget ($\sim$8.2–8.7 G), MoR-Swin-B reaches 83.0% IN-1K Top-1 vs. Swin-S's 83.2% at 8.7 G with roughly $1.4\times$ the throughput; and at a matched accuracy

of ∼83% MoR-Swin-B uses 8.2 G vs. Swin-B's 15.4 G, a 47% reduction. (For reference, MoR-Swin-B's IN-22K→1K transfer accuracy is 84.5%, reported separately in that same table; we do *not* mix this transfer number into the IN-1K Pareto curve.)

2. **Latency frontier**: MoR-Swin occupies the upper-left region of the Acc-vs-Latency plot across all scales, confirming it defines a strictly better Pareto frontier than both Swin and Swin-MoE.
3. **Scaling behavior**: The efficiency advantage grows with model size (Base and Large benefit more from adaptive early-exit than Tiny), because larger models have more redundant computation for simple tokens.

S8. Feature Diversity under Weight Sharing (CKA Analysis)

To verify that weight sharing across recursion steps does not collapse feature diversity, we compute the Centered Kernel Alignment (CKA) between feature representations at different recursion depths and stages.

Setup: We extract features from MoR-Swin-B at each recursion step $k = 1, \dots, N_a$ within each stage, and compute linear CKA between all pairs on 5,000 ImageNet validation images.

Results:

- **Adjacent steps (within stage)**: CKA = 0.85–0.92, indicating gradual refinement rather than identity mapping or collapse.
- **Non-adjacent steps (within stage)**: CKA = 0.70–0.80, confirming progressive transformation.
- **Cross-stage**: CKA = 0.30–0.45, comparable to the cross-layer CKA in standard Swin (0.28–0.42), confirming that hierarchical feature diversity is preserved.
- **Comparison with Swin**: The cross-layer CKA profile of MoR-Swin closely matches that of standard Swin (Pearson $r = 0.94$ between the two CKA matrices), indicating no significant diversity loss.

S9. Measured GPU Memory Usage

We report the actual peak GPU memory (GB) measured via `torch.cuda.max_memory_allocated()` on a single A100-80G, FP16, batch=64.

To avoid conflating the default model with the optional memory-optimized configuration, Table S5 reports peak memory for MoR-Swin-B under all three configurations, and the model-scale comparison is given for the *default* configuration (recursion-level caching only, selective KV caching disabled), which is the one used for all main-text results.

Table S5. Measured peak GPU memory (GB) for MoR-Swin-B under the three configurations, and the default-configuration comparison across scales. "Train" uses batch=64 with gradient checkpointing disabled; "Infer" uses batch=64 in eval mode. Configurations: (R) recursion-level caching; (KV) selective KV caching. The default main-experiment configuration is (R) only. The "Reduction" column is the average peak-memory reduction *relative to the same-scale Swin baseline* (Swin-B for the MoR-Swin-B rows, Swin-S for the MoR-Swin-S row), averaged over the train/infer and 224/512 columns.

Model / configuration	224×224		512×512		Reduction
	Train	Infer	Train	Infer	(avg.)
Swin-B (baseline)	18.2	4.1	52.6	12.8	—
MoR-Swin-B, default recompute (no caching)	15.9	3.6	45.9	11.2	∼13%
MoR-Swin-B, +R (*default main exp.*)	13.6	3.1	39.4	9.6	∼25%
MoR-Swin-B, +R +KV (memory-optimized)	11.0	2.5	31.4	7.7	∼40%
Swin-S (baseline)	12.5	2.8	36.1	8.7	—
MoR-Swin-S (+R, default main exp.)	9.4	2.1	27.1	6.5	∼25%

The default main-experiment configuration therefore achieves ∼25% peak-memory reduction from recursion-level caching alone (releasing inactive-token intermediates). Enabling the optional selective KV caching (reusing first-step KV) removes the repeated KV copies and raises the total to ∼40%, at the cost of ∼0.3% Top-1 accuracy (see the caching trade-off table and the three-configuration memory table in the main text); this memory-optimized configuration is *not* used for any of the reported accuracy results.

S10. Generalization Beyond Swin: Proof-of-Concept

To evaluate whether the MoR mechanism transfers beyond Swin, we apply it to two additional attention-based backbones under identical training protocols (ImageNet-1K, 300 epochs, same augmentation):

Table S6. MoR applied to other backbones (proof-of-concept, ImageNet-1K).

Model	Params (M)	Top-1 (%)	FPS
ViT-B/16	86	81.8	892
MoR-ViT-B/16	45	81.2	1580
MaxViT-S	69	83.5	725
MoR-MaxViT-S	36	82.9	1310

Both backbones achieve ∼48% parameter reduction and ∼1.8× speedup with <0.7% accuracy loss, confirming that MoR's adaptive recursion generalizes to attention-based architectures beyond Swin. Extension to convolution-based backbones (ConvNeXt-V2, InternImage) requires replacing the RWA module with a recursive convolution block; we leave this as future work.

References

1. Chen, R.T.Q.; Rubanova, Y.; Bettencourt, J.; Duvenaud, D. Neural Ordinary Differential Equations. In *Proc. NeurIPS*, 2018.
